# Identification of Nitrogen-Fixing *Bradyrhizobium* Associated With Roots of Field-Grown Sorghum by Metagenome and Proteome Analyses

**DOI:** 10.3389/fmicb.2019.00407

**Published:** 2019-03-12

**Authors:** Shintaro Hara, Takashi Morikawa, Sawa Wasai, Yasuhiro Kasahara, Taichi Koshiba, Kiyoshi Yamazaki, Toru Fujiwara, Tsuyoshi Tokunaga, Kiwamu Minamisawa

**Affiliations:** ^1^Graduate School of Life Sciences, Tohoku University, Sendai, Japan; ^2^Institute of Low Temperature Science, Hokkaido University, Sapporo, Japan; ^3^Earthnote Co., Ltd., Okinawa, Japan; ^4^Graduate School of Agricultural and Life Sciences, The University of Tokyo, Tokyo, Japan

**Keywords:** *Bradyrhizobium*, nitrogen fixation, sorghum, metagenome, proteome

## Abstract

Sorghum (*Sorghum bicolor*) is cultivated worldwide for food, bioethanol, and fodder production. Although nitrogen fixation in sorghum has been studied since the 1970s, N_2_-fixing bacteria have not been widely examined in field-grown sorghum plants because the identification of functional diazotrophs depends on the culture method used. The aim of this study was to identify functional N_2_-fixing bacteria associated with field-grown sorghum by using “omics” approaches. Four lines of sorghum (KM1, KM2, KM4, and KM5) were grown in a field in Fukushima, Japan. The nitrogen-fixing activities of the roots, leaves, and stems were evaluated by acetylene reduction and ^15^N_2_-feeding assays. The highest nitrogen-fixing activities were detected in the roots of lines KM1 and KM2 at the late growth stage. Bacterial cells extracted from KM1 and KM2 roots were analyzed by metagenome, proteome, and isolation approaches and their DNA was isolated and sequenced. Nitrogenase structural gene sequences in the metagenome sequences were retrieved using two nitrogenase databases. Most sequences were assigned to *nifHDK* of *Bradyrhizobium* species, including non-nodulating *Bradyrhizobium* sp. S23321 and photosynthetic *B. oligotrophicum* S58^T^. Amplicon sequence and metagenome analysis revealed a relatively higher abundance (2.9–3.6%) of *Bradyrhizobium* in the roots. Proteome analysis indicated that three NifHDK proteins of *Bradyrhizobium* species were consistently detected across sample replicates. By using oligotrophic media, we purified eight bradyrhizobial isolates. Among them, two bradyrhizobial isolates possessed 16S rRNA and *nif* genes similar to those in S23321 and S58^T^ which were predicted as functional diazotrophs by omics approaches. Both free-living cells of the isolates expressed N_2_-fixing activity in a semi-solid medium according to an acetylene reduction assay. These results suggest that major functional N_2_-fixing bacteria in sorghum roots are unique bradyrhizobia that resemble photosynthetic *B. oligotrophicum* S58^T^ and non-nodulating *Bradyrhizobium* sp. S23321. Based on our findings, we discuss the N_2_-fixing activity level of sorghum plants, phylogenetic and genomic comparison with diazotrophic bacteria in other crops, and *Bradyrhizobium* diversity in N_2_ fixation and nodulation.

## Introduction

Biological N_2_ fixation in non-leguminous plants is required to improve agricultural sustainability by decreasing the global use of synthetic nitrogen fertilizers (Steffen et al., 2015; Yoneyama et al., 2017; Rosenblueth et al., 2018). Diazotrophic endophytes can provide fixed nitrogen to non-leguminous crops (Boddey et al., 2001; Yoneyama et al., 2017). ^15^N-labeling significantly contributed to N_2_ fixation in sugarcane (Yoneyama et al., 1997; Boddey et al., 2001). *Gluconacetobacter* and *Herbaspirillum* were isolated from sugarcane stems as candidate endophytic N_2_-fixing bacteria (Cavalcante and Dobereiner, 1988; James, 2000). Recent metatranscriptome analyses targeting *nifH* (encoding dinitrogenase reductase) suggested that *Bradyrhizobium* members play a role in N_2_ fixation in sugarcane (Thaweenut et al., 2011; Fischer et al., 2012; Rosenblueth et al., 2018). Abundant expression of *Bradyrhizobium* and *Azorhizobium nifH* was also detected in sweet potato stems and tubers (Terakado-Tonooka et al., 2008).

Sorghum [*Sorghum bicolor* (L.) Moench] is a C_4_ plant. Sorghum has little breeding history compared to sugarcane and maize but has the potential for broad agro-ecological adaptation (Khawaja et al., 2014). Sorghum provides grain for use in food and feed, sugary juice for producing syrup or bioethanol and is an excellent fodder (Khawaja et al., 2014). Omics studies of sorghum-associated microbes (Naylor et al., 2017; Xu et al., 2018) showed that drought increased the abundance and activity of monoderm bacteria including *Actinobacteria* in field-grown sorghum and verified that these bacteria contribute to the drought-resistance of sorghum plants. Thus, sorghum root-associated microbiomes play an important role in determining plant fitness.

For nitrogen fixation in sorghum plants, Pedersen et al. (1978) first detected the N_2_-fixing activities of washed root segments and soil cores of grain sorghum in NE, United States, in an acetylene reduction assay. Wani et al. (1984) observed the acetylene-reducing activity (ARA) of intact sorghum plants grown in pots. These studies suggested that sorghum-associated bacteria play a role in N_2_ fixation. Coelho et al. (2008) reported several diazotrophic bacteria (*Paenibacillus, Azohydromonas, Ideonella, Rhizobium*, and *Bradyrhizobium*) in the rhizosphere soils of field-grown sorghum in Brazil based on *nifH* PCR of soil DNA extracts. However, N_2_-fixing bacteria associated with sorghum plant tissues have not been fully explored.

**FIGURE 1:**
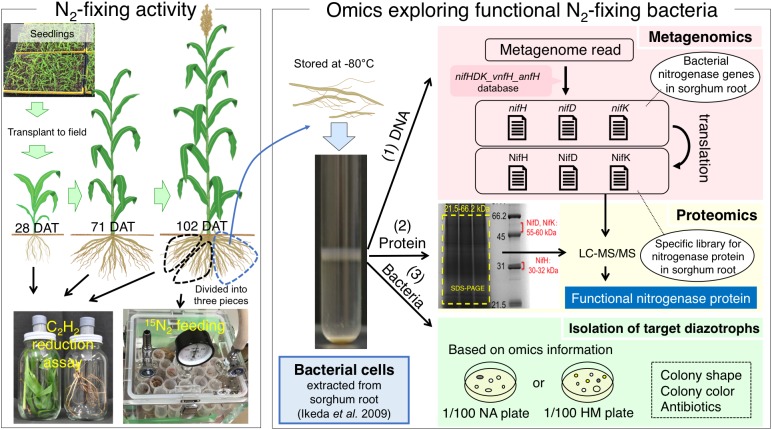
Outline of “omics” strategy used to explore and identify functional N_2_-fixing bacteria associated with sorghum plants. N_2_-fixing activities were monitored in tissues of sorghum at different growth stages by acetylene reduction assay and were directly confirmed in an ^15^N_2_ feeding experiment. Bacteria were extracted from sorghum root tissues with higher N_2_-fixing activities, and their metagenomes (1) and proteomes (2) were analyzed. Functional N_2_-fixing bacteria were isolated from the extracted bacteria (3). DAT = days after transplant.

Recent “omics” approaches have been used to identify and isolate functional diazotrophs in sugarcane (Thaweenut et al., 2011; Fischer et al., 2012), sweet potato (Terakado-Tonooka et al., 2008; Terakado-Tonooka et al., 2013), and paddy rice (Bao et al., 2014, 2016). Particularly, the combination of metagenome and metaproteome analyses based on extracted bacterial cells (EBCs) isolated from plant tissues (Ikeda et al., 2009) revealed type II methanotrophs in paddy rice roots as functional N_2_-fixing bacteria (Bao et al., 2014; Minamisawa et al., 2016). We adopted a similar strategy to identify diazotrophs responsible for N_2_ fixation in field-grown sorghum plants. We identified tissues showing significant N_2_-fixing activity by ARA and ^15^N_2_ fixation, identified functional diazotrophs by proteome analysis of nitrogenase proteins based on metagenomic data, and isolated bacteria with nitrogenase proteins and phylogenetic markers predicted from the omics results (Figure 1). Our results strongly suggest that bradyrhizobia fixed N_2_ in the roots of filed-grown sorghum plants at late growth stages. Because the N_2_-fixing bradyrhizobia in sorghum roots are phylogenetically close to an aquatic legume, *Aeschynomene* (Okubo et al., 2012a), we describe their functional roles.

## Materials and Methods

### Plant Materials and Field Conditions

We used four lines (KM1, KM2, KM4, and KM5) of sorghum developed by Earthnote Co., Ltd. (Okinawa, Japan). KM1 is a late-ripening line with vigorous leaf growth. KM2 is an early-ripening line with lodging resistance and salt tolerance. KM4 and KM5 were pre-selected because of their high (KM4) and low (KM5) N_2_-fixing activities as estimated by the ^15^N dilution method (Lee et al., unpublished).

Seeds were sown in 200-cell plug trays on May 10, 2016. The seedlings were transplanted into a field owned by Earthnote (Fukushima, Japan; 37°30′46.43^′′^140°34′13.7^′′^) on June 6, 2016. The soil had the following chemical properties: pH (H_2_O), 5.9; total C, 13.9 g kg^-1^ dry soil; total N, 0.8 g kg^-1^ dry soil; available phosphorus, 560.4 mg P kg^-1^ dry soil (Truog method). Before transplanting the seedlings, the field was treated with 85 kg N as urea, 84 kg N as controlled-release coated-urea fertilizer (LP100, JCAM Agri. Co., Ltd., Tokyo, Japan), which releases 80% of its total N over 100 days, and 85 kg K_2_O as potassium sulfate per hectare. This is the standard fertilization regime used for sorghum cropping under the local conditions. Each line was planted in three plots, with 50 plants per plot (0.075 m between plants, 1.00 m between rows).

### Outlines of Polyphasic Approach and Experimental Design

Figure 1 shows the outlines of our polyphasic approach based on nitrogen-fixing activity and omics analyses. Because the experimental designs were complicated for respective analyses, we show the detailed sampling strategies used in the polyphasic approach along with the sorghum growth stages in Supplementary Figures S1, S2.

At least three plants were sampled individually from three different field plots or from the seedling trays for transplanting. When the sorghum seedlings were transplanted into the field, six seedlings were randomly selected from the plug trays [0 days after transplanting (DAT)], which were subjected to the acetylene reduction assay (Supplementary Figure S1A) as described below. At 28 DAT and 71 DAT, field-grown sorghum plants were harvested from three plots individually, and each plant tissue including the leaves, stems, and roots were subjected to the acetylene reduction assay (Supplementary Figure S1B). At 102 DAT, three plants were harvested from three plots individually, and the roots were washed with tap water, weighted, and further subdivided into three parts for the acetylene reduction assay, ^15^N_2_-feeding analysis, and omics/isolation analysis (metagenome, proteome, bacterial isolation) (Supplementary Figure S2 and Figure 1). Thus, the acetylene reduction assay, ^15^N_2_-feeding experiment, and omics analyses (metagenome and proteome) were performed based on three biological replicates.

### Acetylene Reduction Assay ofField-Grown Sorghum

Nitrogen-fixing activity was examined by the acetylene reduction assay (Schollhorn and Burris, 1967) at four growth stages: 0, 27, 71, and 102 DAT. Sorghum plants were maintained in the vegetative stage until 71 DAT, while the plants generally moved to the reproductive stage with ears at 102 DAT (Figure 1). One or two healthy plants were randomly sampled (Supplementary Figures S1, S2). The tissues to be used for ARA were determined according to the growth stage of the plant (Figure 1): 0 DAT, whole seedling; 28 DAT, shoot and root; 71 DAT, leaf, stem, and root; and 102 DAT, root. At 0 and 28 DAT, two plants were harvested, one of which was used for acetylene reduction with acetylene, while the others were used for control analysis without acetylene (Supplementary Figure S1). At 71 and 102 DAT, one plant was divided equally into two pieces of similar weight and structure; one piece was used to analyze acetylene reduction with acetylene and the other was used for control analysis without acetylene (Supplementary Figures S1, S2).

At 0 DAT, seedling roots were washed in tap water to remove adhering soil and placed in a 100-mL vial with a butyl rubber septum (SVG-100, Nichiden-Rika Glass Co., Ltd., Kobe, Japan) (Figure 1). At 27, 71, and 102 DAT, each tissue was placed in a 900-mL glass jar with a modified lid fitted with a butyl rubber W-plug (W-24T, Taiyo Kogyo, Tokyo, Japan) (Figure 1).

Acetylene gas (99.9999% vol/vol purity; Toho Acetylene Co., Tokyo, Japan) was injected to provide a concentration of 10% (vol/vol), and the vial or jar was incubated for 1 day at ambient temperature. We drew 20 mL of headspace gas in a sampling syringe and stored it in a vacuumed gas vial (SVG-5; Nichiden-Rika Glass Co.) until analysis. Gas samples (0.5 mL) were analyzed to determine their ethylene concentrations with a Shimadzu GC-18A gas chromatograph equipped with a flame ionization detector and Porapak R column as described previously (Elbeltagy et al., 2001).

### ^15^N_2_ Feeding of Field-Grown Sorghum

At 102 DAT, the divided roots from the same plant used for ARA were introduced into a gas chamber with a sampling port (Figure 1 and Supplementary Figure S2). The gas phase in the chamber was replaced with 32% (v/v) ^15^N_2_ (99.4 atom % ^15^N; Shoko, Tokyo, Japan)/20% O_2_/48% Ar. The negative control included air gas containing atmospheric N_2_ [natural abundance of ^15^N (0.366 atom %)]. After static incubation in the chamber at 25°C for 23 h in the dark, the root samples were dried at 80°C for 3 days and then powdered in a Multi-Beads Shocker (Yasui Kikai, Osaka, Japan). To determine the level of ^15^N_2_ fixation in the roots, we analyzed ^15^N and total N contents of the powdered root tissues with an elemental analyzer/isotope ratio mass spectrometer (Flash EA1112-Delta V Advantage ConFlo IV System; Thermo Fisher Scientific, Waltham, MA, United States).

### Bacterial Cell Extraction and Metagenome Analysis

Root tissues of the same plant used for the ARA and ^15^N_2_-feeding experiments were stored at -80°C for metagenome analysis (Figure 1 and Supplementary Figure S2). EBCs, including both endophytes and epiphytes, were directly prepared from ∼100 g of root tissues as previously reported (Ikeda et al., 2009). The extraction of EBC allowed for the elimination of plant organelles and plant genomic DNA. Metagenomic DNA was prepared from the bacterial cells using an IsoPlant II kit (Nippon Gene, Tokyo, Japan) with bead beating treatment. In the first step, 0.5 g of bacterial cells were disrupted by bead beating in a Lysing Matrix B tube (MP Biomedicals, Santa Ana, CA, United States) with Solution I (Extraction Buffer) from the IsoPlant II kit. Bead beating was performed using a FastPrep FP100A (MP Biomedicals) at 5.5 m s^-1^ for 30 s. Other steps were conducted according to the manufacturer’s instructions, and the DNA preparation was stored at -20°C until use.

DNA libraries were prepared using the Illumina TruSeq DNA library preparation kit v. 2 (Illumina, San Diego, CA, United States). The size and quality of the libraries were assessed using a BioAnalyzer High Sensitivity Chip, and the average fragment size was around 700 base pairs (bp). Next, 250-bp paired-end libraries were sequenced on an Illumina MiSeq sequencer. The quality of the sequence data was checked by using the FastQC v. 0.10.1 software^1^. Reads were trimmed using Prinseq-lite 0.20.4 (Schmieder and Edwards, 2011) with the following settings: -trim_left 20 -trim_right 5 -trim_qual_right 30 -min_qual_mean 20 -min_len 70.

### Extraction of Structural Genes of Nitrogenase

To extract *nifH*, we first assigned the metagenomic reads in each sample according to the results of BLAST analysis against the *nifH* database (Gaby and Buckley, 2014) with a threshold identity of 90%. The *nifH* database developed by Gaby and Buckley (2014) contains 32,954 nitrogenase reductase genes sequences including *anfH*, an alternative nitrogenase that is a paralog of *nifH*, and *vnfH*, a V–Fe alternative nitrogenase. Next, we used candidate reads in BLAST searches against the NCBI NR database with an *E*-value threshold of 10^-10^ and removed false *nifH* reads by manual inspection of the BLAST results. *nifH* genes were taxonomically assigned based on the best BLAST hit and counted. The retrieved reads were translated into amino acid sequences and incorporated into our database for proteome analysis with taxonomic information. If a best-hit was part of a genome sequence, *nifD* and *nifK* were picked up and used for database construction as described below.

To extract *nifD* and *nifK*, we modified the *nifD* and *nifK* database of Gaby and Buckley (2014), which contains only 268 and 315 sequences, respectively. To enrich the *nifDK* database, sequences were collected from (i) NCBI databases matched with respective keywords (“nitrogenase molybdenum-iron,” “*nifD*,” or “*nifK*”) and (ii) *nifD* and *nifK* encoded on genome sequences of *nifH* best-hits. The metagenomic reads in each sample were assigned according to the BLAST analysis against the *nifD* and *nifK* database with a threshold identity of 80% in-house. Next, the candidate reads of *nifD* and *nifK* from the metagenomic data were subjected to BLAST searches against the NCBI NR public database with an *E*-value threshold of 10^-10^, and false *nifD* or *nifK* reads were removed by manual inspection of the BLAST results. The resulting DNA sequences of *nifD* and *nifK* were taxonomically assigned based on the best BLAST hit, translated into amino acid sequences, and incorporated in our nitrogenase database for proteome analysis with taxonomic information.

### Community Analyses of Sorghum Root Microbes

Amplicon sequencing of 16S rRNA and metagenomics analysis targeting 31 AMPHORA genes (Wu and Eisen, 2008), which are housekeeping genes that are mostly present in single copies, were performed to analyze the bacterial community associated with the sorghum roots.

A partial bacterial 16S rRNA gene fragment was amplified by two-step PCR. First, a partial sequence of the V4 variable region was amplified with simultaneous addition of sequencing priming sites. The target region was amplified using primers 515F (5′-ACACTCTTTCCCTACACGACGCTCTTCCGATCTGTGCCAGCMGCCGCGGTAA-3′) and 806R (5′-GTGACTGGAGTTCAGACGTGTGCTCTTCCGATCTGGACTACHVGGGTWTCTAAT-3′) (Caporaso et al., 2011). In the second round of PCR, the first-round PCR products were amplified using the above sequencing priming sites as PCR priming sites by adding dual-index tag sequences and flow cell binding sites of the Illumina adapter. The primer sequences were 5′-AATGATACGGCGACCACCGAGATCTACAC–Index2–ACACTCTTTCCCTACACGACGC-3′ (forward) and 5′-CAAGCAGAAGACGGCATACGAGAT–Index1–GTGACTGGAGTTCAGACGTGTG-3′ (reverse). Each 20-μL PCR mixture contained 0.2 μL TaKaRa ExTaq HS DNA polymerase (TaKaRa Bio, Shiga, Japan), 2.0 μL of buffer (10 × Ex buffer), 1.6 μL of 2.5 mM dNTP mix, 1.0 μL of each forward and reverse primer (10 μM), and 1 μL of template DNA. First-round PCR conditions were as follows: 94°C for 2 min, followed by 25 cycles of 94°C for 30 s, 50°C for 30 s, and 72°C for 30 s. The PCR products were purified using an Agencourt AMPure XP purification system (Beckman Coulter, Brea, CA, United States) following the manufacturer’s instructions. Second-round PCR conditions were as follows: 94°C for 2 min; 12 cycles of 94°C for 30 s, 60°C for 30 s, and 72°C for 30 s; and a final 72°C for 5 min. The purified tag-indexed PCR products were quantified by using a Qubit 2.0 Fluorometer and dsDNA HS Assay Kit (Life Technologies, Carlsbad, CA, United States). Samples were then pooled in equal amounts and sequenced by using a 250-bp paired-end sequencing protocol with the MiSeq Sequencing Reagent Kit v. 2 (Illumina) at Bioengineering Lab Co., Ltd., (Kanagawa, Japan). Sequence reads of all samples were processed using QIIME2 (Caporaso et al., 2010). In summary, assembly of pair-end reads was performed with default settings and then primer removal, quality control, and chimeric sequence trimming were performed using dada2 (Callahan et al., 2016). Taxonomic classification was assigned using a SILVA 16S rRNA reference alignment [Release 132 (Quast et al., 2013)]. Each sample contained 0.3–1.7% of plant organelle reads. Therefore, all sequences classified as chloroplast or mitochondria were removed.

A universal marker set of 31 protein-encoding phylogenetic marker genes was extracted from the metagenome data of KM1 and KM2 using AMPHORA2 with default settings (Wu and Scott, 2012).

### Proteome Analysis

Proteins were extracted from the EBCs from the roots, and ∼50 μg of sample was separated by 12.5% sodium dodecyl sulfate-polyacrylamide gel electrophoresis (SDS–PAGE) and stained with Coomassie blue. Gel strips containing proteins ranging from 21.5 to 66.2 kDa (NifHDK protein sizes) were excised (Figure 1). Proteome analysis was performed as described previously (Kasahara et al., 2012; Bao et al., 2014). Briefly, the gel lanes were cut into strips of approximately 1 mm, and the gel strips were digested with trypsin. Nano-liquid chromatography (LC)–electrospray ionization–tandem mass spectrometry (MS/MS) analysis of the peptide mixtures was performed with an LTQ ion-trap MS (Thermo Fisher Scientific) coupled with a multidimensional high-performance LC (HPLC) Paradigm MS2 chromatograph (AMR, Inc., Tokyo, Japan) and nanospray electrospray ionization device (Michrom Bioresources, Inc., Auburn, CA, United States). The MS/MS data were searched against the protein database constructed in this study using Mascot program ver. 2.4 (Matrix Science, London, United Kingdom).

### Isolation of Bradyrhizobia From Sorghum Roots

The bacterial cells extracted from the roots of lines KM1 and KM2 were pre-incubated at 28°C for 6 h in HM salt medium (Cole and Elkan, 1973) supplemented with 0.1% (w/v) arabinose and 0.025% (w/v) yeast extract to activate the bacterial cells for subsequent plating. These samples were serially diluted with sterilized water and plated on two types of agar plates, 1/100-strength nutrient medium (BD Biosciences, Franklin Lakes, NJ, United States) containing 1.5% agar and 10 mg L^-1^ polymixin B or 1/100-strength HM salt medium containing 1.5% agar and 10 mg L^-1^ polymixin B. This isolation strategy was used because *Bradyrhizobium* species are oligotrophic slow-growing bacteria (Okubo et al., 2012b, 2013) and resistant to polymyxin B (Hirayama et al., 2011; Piromyou et al., 2015a). After 10 days of cultivation at 28°C, slow-growing white colonies were picked up and re-streaked on the same types of agar plates for the first cultivation without polymixin B. This step was repeated one more time. Cell lysates of the resulting isolates were used to amplify the 16S rRNA gene by PCR as described previously (Itakura et al., 2009).

### Phylogenetic Analyses of Bradyrhizobial Isolates

The 16S rRNA gene regions were amplified with Blend Taq polymerase (Toyobo, Osaka, Japan) and sequenced by direct PCR using universal forward (27f) and reverse (1492r) primers (Lane, 1991). The PCR conditions were as follows: preheating at 94°C for 2 min; 30 cycles of denaturation at 94°C for 30 s, annealing at 55°C for 30 s, and extension at 72°C for 90 s; extension at 72°C for 60 s; a final extension at 72°C for 5 min, and cooling at 12°C. For direct PCR sequence analysis with the 27f primer, an ABI Prism 3130xl genetic analyzer was used with a BigDye Terminator v. 3.1 Cycle Sequencing Ready-Reaction Kit (Applied Biosystems, Foster City, CA, United States). Phylogenetic trees of the gene sequences were constructed in MEGA v. 7.0 software (Tamura et al., 2011) by using the neighbor-joining method (Saitou and Nei, 1987).

### Mapping of Shotgun Sequences

Genomic DNA of bradyrhizobial isolates TM122 or TM124 was extracted with an Illustra Bacteria GenomicPrep Mini Spin Kit (GE Healthcare, Little Chalfont, United Kingdom). DNA libraries were prepared with a Nextera DNA Sample Preparation Kit and sequenced on an MiSeq system using a MiSeq Reagent Nano Kit v. 2 (500 cycles) (both Illumina). Raw reads were trimmed in CLC Genomics Workbench v. 7.5.1 software (CLC Bio, Aarhus, Denmark) with the following parameters: ambiguous limit, 2; quality limit, 0.05; number of 5′ terminal nucleotides, 20; number of 3′ terminal nucleotides, 5; minimum number of nucleotides in reads, 70.

Shotgun sequence reads of the isolates were mapped to a reference genome of *B. diazoefficiens* USDA110^T^ (Kaneko et al., 2002). Mapping was performed in CLC Genomics Workbench software with the following parameters: mismatch cost, 2; insertion cost, 3; deletion cost, 3; length fraction, 0.9; similarity fraction, 0.9. The shotgun sequences of TM122 and TM124 were assembled in CLC Genomics Workbench software with default parameters. The contigs containing *nif* genes were annotated by using the Microbial Genome Annotation Pipeline (MiGAP^2^).

### Acetylene Reduction Assay of Isolates

*Bradyrhizobium* sp. TM122, and TM124 were pre-cultured in HM medium (Cole and Elkan, 1973) with shaking at 28°C. Five milliliters of the exponential growing culture were collected by centrifugation (13,000 × *g*, 3 min, 4°C) and washed twice with sterilized water. The cell density was adjusted to 10^7^ cells/mL, and 1 mL of the cell suspension was inoculated into 7 mL of Rennie semi-solid medium (5 g of sucrose, 5 g of mannitol, 0.5 mL of sodium lactate, 0.8 g of K_2_HPO_4_, 0.2 g of KH_2_PO_4_, 0.1 g of NaCl, 28 mg of Na_2_FeEDTA, 25 mg of Na_2_MoO ⋅ 4H_2_O, 100 mg of yeast extract, 0.2 g of MgSO_4_ ⋅ 7H_2_O, 0.06 g of CaCl_2_, 5 μg of biotin, 10 μg of *para*-aminobenzoic acid, and 0.2 g of noble agar per L) (Rennie, 1981) in a 28-mL test tube, which was capped with a butyl rubber W-plug. After the test tubes were incubated at 28°C for 3 days, 10% (v/v) of the gas phase was replaced with acetylene (99.9999% vol/vol purity; Toho Acetylene Co., Tokyo, Japan). The samples were re-incubated at 28°C without shaking for 3 days. The gas phase (0.5 mL) was sampled to determine the concentration of ethylene by a gas chromatograph as described above.

### Acetylene Reduction Assay of Sorghum Seedling Inoculated With Isolates

Sorghum seeds of KM2 were shaken in 0.5% NaOCl solution for 1 min and then washed five times with sterile distilled water. Two sterilized seeds were planted in a Leonard’s jar containing sterilized vermiculite (Inaba et al., 2012) and plant nutrient solution (Mae and Ohira, 1981) with 0.05 mM of NH_4_NO_3_ under aseptic conditions (Supplementary Figure S3). A cell suspension of TM122 or TM124 was added to the sorghum seeds at a concentration of 10^7^ cells per seed.

The plants were grown in a growth cabinet (LH300; NK System Co., Ltd., Osaka, Japan) that provided 65 mol photons m^-2^ s^-1^ of photosynthetically active radiation (400–700 nm) under a daily cycle of 16 h of light and 8 h of dark at 25°C. After 7 days, the seedlings were thinned out to one plant. At 28 days after inoculation, the roots were carefully harvested and then transferred into a 100-mL vial with a butyl rubber septum (SVG-100, Nichiden-Rika Glass Co., Ltd.). Acetylene (10 mL) was introduced into the bottles, which were incubated for 24 h at 25°C in the dark.

### Accession Numbers

DNA sequences obtained by the metagenomic analysis and 16S rRNA gene amplicon, TM122 genome, and TM124 genome were deposited under the accession numbers DRA006465, DRA006466, DRA006492, and DRA006493 in the DDBJ Sequence Read Archive, respectively. The sequences of 16S rRNA genes of bacterial isolates were deposited in DDBJ/EMBL/GenBank under the accession numbers LC367220–LC367221 and LC433572–LC433596, respectively.

## Results

### Acetylene Reduction Assay of Field-Grown Sorghum

N_2_-fixing activities were monitored at different growth stages for four sorghum lines (KM1, KM2, KM4, and KM5) by an acetylene reduction assay in closed bottles (Figure 1 and Supplementary Figures S1, S2). Because sorghum tissues emit ethylene as a plant hormone, we calculated the ARA of bacterial nitrogenase as the difference in the rate of ethylene production in the presence and absence of acetylene (Supplementary Table S1).

We detected no ARA in the seedlings at 0 DAT or in the shoots and roots at 28 DAT (Table 1). However, we detected significant ARA values (KM1, 36.1 nmol plant^-1^ h^-1^, KM2, 52.6 nmol plant^-1^ h^-1^) at 71 DAT in the washed roots (Table 1). At 102 DAT, the roots of all lines showed significantly higher ARA values than at 71 DAT. Particularly, the roots of KM1 showed the highest ARA (585.8 nmol plant^-1^ h^-1^), followed by line KM2 (332.5 nmol plant^-1^ h^-1^). The ARA of KM5 roots (6.7 nmol plant^-1^ h^-1^) was significantly lower than that of KM1 (Table 1), indicating that root ARA depends on the sorghum genotype.

**Table 1:** Acetylene-reducing activity (ARA) of four sorghum lines (KM1, KM2, KM4, and KM5) at each growth stage*^a^*.

DAT	Tissue	Acetylene-reducing activity (ARA) (nmol C_2_H_4_ plant^-1^ h^-1^)							
		
		KM1		KM2		KM4		KM5	
0	Seedling	0.0 ± 0.0	C	0.1 ± 0.1	C	0.5 ± 0.4	C	-0.1 ± 0.0	C
28	Shoot	0.1 ± 0.3	C	-0.9 ± 0.2	C	0.1 ± 0.6	C	0.3 ± 0.6	C
28	Root	-0.1 ± 0.0	C	0.2 ± 0.1	C	0.0 ± 0.2	C	0.1 ± 0.0	C
71	Leaf	0.4 ± 0.4	C	1.6 ± 0.4	C^†^	0.2 ± 0.0	C	0.2 ± 0.1	C
71	Stem	1.4 ± 0.6	C	0.4 ± 0.5	C	0.7 ± 0.4	C	0.1 ± 0.5	C
71	Root	36.1 ± 10.9	C^†^	52.6 ± 15.6	C^†^	25.1 ± 9.2	C	1.7 ± 0.7	C
102	Root	585.8 ± 100.0	A^‡^	332.5 ± 21.8	B^‡^	292.1 ± 73.3	B^†^	6.7 ± 1.5	C^†^


*^*a*^*Data are mean ± SEM (*n* = 3). Daggers indicate significant increase in ethylene production in the presence of acetylene (positive acetylene-reducing activity) compared to natural ethylene production in plant tissues in the absence of acetylene (Welch’s *t*-test, ^†^*P* < 0.05; ^‡^*P* < 0.01; Supplementary Table S1). DAT = days after transplanting. Means with the same letter were not significantly different by Tukey’s HSD test (*P* < 0.05).

### ^15^N_2_-Feeding Experiment of Field-Grown Sorghum

The above results suggest that N_2_ fixation in the roots is high in late growth stages of some sorghum lines. To confirm N_2_ fixation more directly, we conducted ^15^N_2_-feeding experiments using the roots at 102 DAT (Figure 1 and Supplementary Figure S2). When the roots were exposed to ^15^N_2_ for 23 h, ^15^N was apparently incorporated into the root tissues inhabited by bacteria including diazotrophs, indicating that the bacteria fixed N_2_ (Table 2). We calculated the ^15^N_2_-fixing activity from the ^15^N concentration (atom% excess) and total N amount (biomass × N content) (Table 2). Although the four lines showed ^15^N_2_-fixation, the highest activity was detected in the roots of KM1 (102.8 μg N plant^-1^ day^-1^), which was significantly higher than the values in the other lines (Table 2). KM2 showed the second highest activity (40.7 μg N plant^-1^ day^-1^).

**Table 2:** Incorporation of ^15^N from ^15^N_2_ into sorghum roots at 102 days after transplant*^a^*.

Sorghum line and gas phase	^15^N concentration			Root biomass		Root N content	N_2_ fixation	
				
	‰	Atom%	Atom% excess (a)	Root dry wt (g) plant^-1^ (b)		% of total dry wt (c)	μg-N plant^-1^ day^-1^ (d)	
KM1								
^15^N_2_	57.0 ± 14.0	0.387 ± 0.005	0.021 ± 0.005	88.7 ± 17.9	A	0.54 ± 0.06	102.8 ± 25.6	A
None	3.6 ± 1.4	0.368 ± 0.000														
KM2																				
^15^N_2_	50.3 ± 23.0	0.385 ± 0.008	0.019 ± 0.008	46.5 ± 16.3	AB	0.57 ± 0.04	40.7 ± 9.0	B
None	1.3 ± 0.9	0.367 ± 0.000														
KM4																				
^15^N_2_	41.7 ± 6.7	0.382 ± 0.002	0.016 ± 0.002	24.4 ± 3.6	B	0.68 ± 0.08	26.1 ± 3.2	B
None	1.5 ± 0.1	0.367 ± 0.000														
KM5																				
^15^N_2_	34.4 ± 12.1	0.379 ± 0.004	0.013 ± 0.004	1.6 ± 0.3	B	0.61 ± 0.02	1.3 ± 0.5	B
None	1.2 ± 1.3	0.367 ± 0.000														


*^a^Data are mean ± SEM (*n* = 3). DAT = days after transplant. N_*2*_ fixation was based on differences in ^*15*^N concentrations between root systems with and without ^*15*^N_*2*_ (None) (see text). Means with the same letter were not significantly different by Tukey’s HSD test (*P* < 0.05). N_*2*_ fixation rate (d) was respectively calculated for three datasets of each sorghum line according to the following formula: d (μg-N plant^-*1*^ day^-*1*^) = (b × c × 10^-*2*^) × 10^*6*^ × a (atom%)/99.4 (atom%) × 24 h/23 h.*

### Comparison Between ARA and ^15^N_2_-Feeding Experiments

ARA values were converted to gram N basis to compare the results of the ARA and ^15^N_2_-feeding experiments (Table 3). N_2_ fixation values in KM1 roots reached a maximum at 102 DAT and were nearly identical between the ARA and ^15^N_2_-feeding methods (98.4–102.8 μg N plant^-1^ day^-1^; Table 3). N_2_ fixation values in KM2 and KM4 roots were lower in the ^15^N_2_ feeding method than in ARA measurement. KM5 roots consistently showed the lowest values (∼1 μg N plant^-1^ day^-1^) in both methods. Thus, we focused on the root microbiomes of KM1 and KM2 at 102 DAT for subsequent metagenome, proteome, and isolation analyses (Figure 1) because their N_2_-fixing activities were the highest (Table 2).

**Table 3:** Comparison of acetylene-reducing activity and ^15^N_2_ feeding methods of evaluating N_2_-fixing activity in roots of four sorghum lines at 102 days after transplant*^a^*.

Sorghum line	N_2_ fixation from acetylene-reducing activity (ARA)			N_2_ fixation from ^15^N_2_ feeding	^15^N/ARA ratio
			
	nmol C_2_H_4_ plant^-1^ h^-1^	nmol N_2_ plant^-1^ day^-1^	μg N plant^-1^ day^-1^	μg N plant^-1^ day^-1^	
KM1	585.8 ± 100.0	3515 ± 600	98.4 ± 16.8	102.8 ± 25.6	1.18 ± 0.41
KM2	332.5 ± 21.8	1995 ± 131	55.9 ± 3.7	40.7 ± 9.0	0.73 ± 0.16
KM4	292.1 ± 73.3	1753 ± 440	49.1 ± 12.3	26.1 ± 3.2	0.59 ± 0.11
KM5	6.7 ± 1.5	40 ± 9	1.1 ± 0.3	1.3 ± 0.5	1.21 ± 0.41


*^a^Average values of acetylene-reducing activity and ^*15*^N_*2*_ feeding with SEM (*n* = 3) are derived from Tables 1, 2, respectively. For calculations of nmol N_*2*_ fixation from ARA, the molar conversion factor of 4 from ARA to N_*2*_ fixation was based on equations 1 and 2 below. ^*15*^N/ARA ratio indicates the ratio of N_*2*_ fixation estimated by ARA and ^*15*^N_*2*_ feeding experiments. N_*2*_ + 8H^+^ + 8e^-^ + 16Mg-ATP → 2NH_*3*_ + H_*2*_ + 16Mg-ADP + 16 Pi (Eq. 1 for physiological reaction of nitrogenase). 4C_*2*_H_*2*_ + 8H^+^ + 8e^-^ + 16Mg-ATP → 4C_*2*_H_*4*_ + 16Mg-ADP + 16 Pi (Eq. 2 for acetylene reduction by nitrogenase).*

### Metagenome Analysis

We extracted bacterial cells from the roots of KM1 and KM2 with three biological replicates at 102 DAT. The metagenomes of the six DNA samples were sequenced by MiSeq. The metagenomic data comprised 4.3–5.4 × 10^6^ reads and 196–208-bp average lengths per DNA sample (Supplementary Table S2).

**FIGURE 2:**
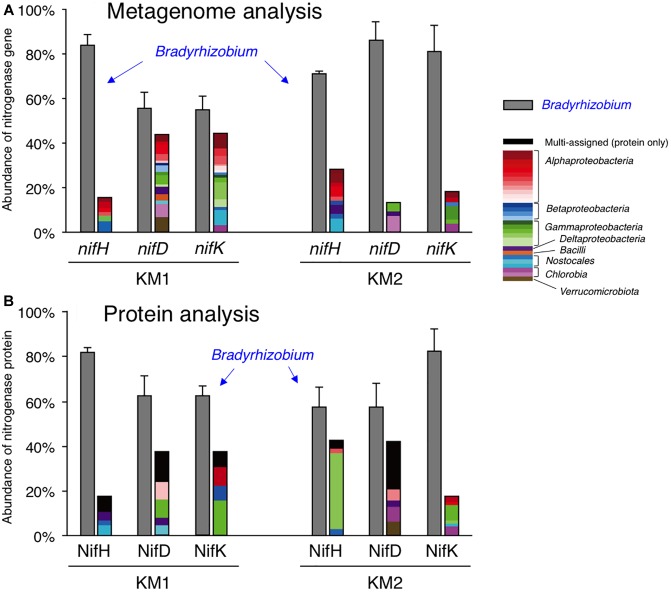
Relative abundance of *nifHDK* reads **(A)** and NifHDK peptides **(B)** of respective bacterial genera. Data represent the average of three biological replications. The color of bars indicates assigned taxonomy. Gray indicates *Bradyrhizobium*. Black in panel B indicates multi-assigned peptides. Bar indicates standard error of three biological replicates.

By using a *nif* database (see section “Materials and Methods”), we retrieved genes encoding nitrogenase reductase (NifH) and dinitrogenase (NifDK) from the six datasets. Most nitrogenase genes were proteobacterial *nifHDK* genes (Supplementary Table S3 for KM1, Supplementary Table S4 for KM2) and did not include *vnfH, anfH*, or archaeal nitrogenase genes. At the genus level, *Bradyrhizobium nifHDK* genes were most abundant (Figure 2A, 55–86% in each nitrogenase gene of KM1 and KM2), and three *nif* structural genes (*nifHDK*) of *Bradyrhizobium* were consistently detected among the six datasets including sorghum lines KM1 and KM2 (Figure 2A and Supplementary Table S5). In contrast, *nifHDK* genes of other genera showed relatively low abundance (Figure 2A, 1–7% in each nitrogenase gene of KM1 and KM2), with parts of three *nif* structural genes (Supplementary Table S5). For example, *nif* structural genes from *Azorhizobium* were exclusively found in *nifDK* genes from the sorghum line KM1 (Supplementary Table S5).

BLAST analysis indicated that most bradyrhizobial *nifHDK* genes belonged to the non-nodulating *Bradyrhizobium* sp. S23321 (AP012279) (Okubo et al., 2012a) and photosynthetic *B. oligotrophicum* S58^T^ (AP012603) (Okubo et al., 2013) (Supplementary Tables S6, S7). Among the *Bradyrhizobium nifHDK* reads, S23321 *nifH* genes accounted for 40% of the *nifH* reads from KM1 reads and 45% of the *nifH* reads from KM2 reads. S58^T^ *nifH* genes accounted for 17% and 21%, respectively (Supplementary Tables S6, S7). Abundant *nifDK* reads were also observed in S23321 (31–37%) and S58 (11–29%) among *Bradyrhizobium nifDK* reads of sorghum lines KM1 and KM2 (Supplementary Tables S6, S7). These results suggest that *Bradyrhizobium*-homologous *nifHDK* genes are abundant in the KM1 and KM2 root microbiomes. The expression of nitrogenase genes is regulated by oxygen and nitrogen, as microbial N_2_ fixation requires a large amount of energy (Yoneyama et al., 2017; Rosenblueth et al., 2018). Thus, we examined which *nifHDK* genes were expressed in the plant environments by determining their resulting protein levels.

### Proteome Analysis

We analyzed the proteomes of EBCs from the roots of KM1 and KM2 at 102 DAT (Figure 1). The proteins were separated by SDS–PAGE, and we excised gel strips with protein sizes ranging from 21.5 to 66.2 kDa because the estimated sizes of NifH as well as NifD and NifK (Boyd and Peters, 2013; Gaby and Buckley, 2014) were 30–32 and 55–60 kDa, respectively (Figure 1). First, we constructed a database of NifHDK peptides based on the DNA sequences of *nifHDK* genes from our metagenome data (Data Set 1). Next, amino acid sequences of the peptide generated by proteome analysis (Data Set 2) were assigned based on 100% identity (Data Set 2) in the NifHDK database (Data Set 1). The results were summarized at the genus level (Figure 2B and Table 4), which revealed that the NifHDK peptides were from 15 genera and 1 family of *Alpha*-, *Beta*-, *Gamma*-, and *Delta*-*proteobacteria* and cyanobacteria in the root microbiomes. Among them, *Bradyrhizobium* NifHDK peptides were simultaneously detected in all three sampling replicates with an apparently higher abundance than those of other genera (Table 4 and Supplementary Table S8). At the strain level, the bradyrhizobial NifHDK peptides (Table 4) were heavily assigned to NifHDK of *Bradyrhizobium* sp. S23321 and *B. oligotrophicum* S58 in sorghum lines KM1 and KM2, although the NifD peptide of *B. oligotrophicum* S58 was not detected in sorghum line KM2 (Supplementary Table S9). In contrast, NifH peptides of *Kosakonia* of *Gammaproteobacteria* were abundantly detected in KM2, whereas the corresponding NifDK peptides were not abundant (Table 4 and Supplementary Table S8). Three peptides (NifHDK) were not simultaneously detected in non-bradyrhizobia (Table 4).

**Table 4:** Total number of NifHDK peptides in root microbiomes of sorghum lines KM1 and KM2*^a^*.

Class	Genus	KM1			KM2		
			
		NifH	NifD	NifK	NifH	NifD	NifK
*Alphaproteobacteria*	*Bradyrhizobium*	32^∗∗^	17^∗∗^	13^∗∗^	23^∗∗^	21^∗∗^	27^∗∗^
*Alphaproteobacteria*	*Hartmannibacter*			2^∗^			2
*Alphaproteobacteria*	*Methylobacterium*						1
*Alphaproteobacteria*	*Rhizobium*	2			1		
*Alphaproteobacteria*	*Rhodospirillum*		2^∗^			2^∗^	
*Betaproteobacteria*	*Pseudodesulfovibrio*			1			
*Gammaproteobacteria*	*Klebsiella*		2^∗^	4^∗^			4^∗^
*Gammaproteobacteria*	*Kosakonia*			1	15^∗∗^	1	
*Deltaproteobacteria*	*Desulfovibrio*		1			1	
*Nostocales*	*Anabaena*	1			1		
*Nostocales*	*Calothrix*		1				
*Nostocales*	*Nostoc*	2					1
*Chlorobia*	*Chlorobaculum*					1	2^∗^
*Verrucomicrobia*	*Opitutaceae*					3	
Others		2	4	1		3	
	Total	39	27	21	40	31	38


*^*a*^Data obtained from three biological replications. Asterisks indicate that each nitrogenase protein was detected in ^∗∗^three and ^∗^two replicates. The original data with three replications of sorghum plants are shown in Supplementary Table S8.*

Therefore, our culture-independent results indicate that *Bradyrhizobium* species fixed N_2_ at the late growth stage of sorghum lines KM1 and KM2 under field conditions. Particularly, close relatives of *Bradyrhizobium* sp. S23321 and *B. oligotrophicum* S58 are likely crucial candidates as functional diazotrophs in field-grown sorghum roots according to our proteome results.

### Microbial Community of Sorghum Root

**FIGURE 3:**
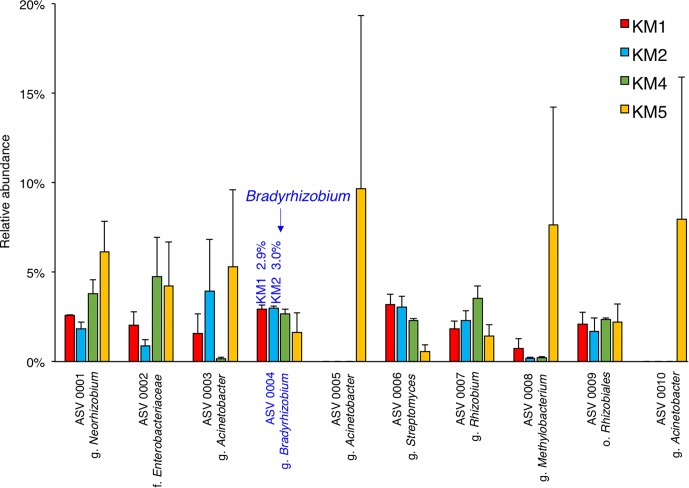
Relative abundance of ten most abundant ASV (amplicon sequence variants) in sorghum lines KM1, KM2, KM4, and KM5 by amplicon analysis of 16S rRNA genes. The ASVs were identified at the lowest possible classification, where o. is order, f. is family and g. is genus. Bar indicates standard error of three biological replicates.

We sequenced amplicons of 16S rRNA genes to evaluate the relative abundance of *Bradyrhizobium* members in the root microbiomes of the four sorghum lines at 102 DAT. Taxonomic classification represented Amplicon Sequence Variants (ASVs) generated by dada2 (Callahan et al., 2016) in QIIME2 (Caporaso et al., 2010). The relative abundances of *Bradyrhizobium* (ASV 0004) in KM1 and KM2 root microbiomes were 2.9 and 3.0%, respectively (Figure 3). Phylogenetic analysis of our metagenomic data using 31 AMPHORA genes also showed a similar relative abundance of *Bradyrhizobium* of KM1 and KM2 (3.6%) (Supplementary Figure S4). These results suggest that *Bradyrhizobium* species are consistent members of the root microbiomes of sorghum lines KM1 and KM2.

### Isolation of Bradyrhizobia From Sorghum Roots

Because horizontal transfer of *nif* genes among several linages of bacteria (Boyd and Peters, 2013), and even within *Bradyrhizobium* (Okubo et al., 2016), has been suggested, *Bradyrhizobium* may not always harbor bradyrhizobial *nif* genes. Therefore, we isolated bradyrhizobia from the sorghum roots on two types of oligotrophic media (1/100-strength nutrient agar medium or 1/100-strength HM agar medium) containing polymixin B. Twenty-eight slow-growing white colonies were isolated, eight of which were identified as *Bradyrhizobium* based on 16S rRNA gene sequencing (Supplementary Table S10). The other 20 isolates belonged to *Ancylobacter, Boseae, Sphingobium, Mesorhizobium, Deinococcus, Mycobacterium*, and *Terrabacter*.

**FIGURE 4:**
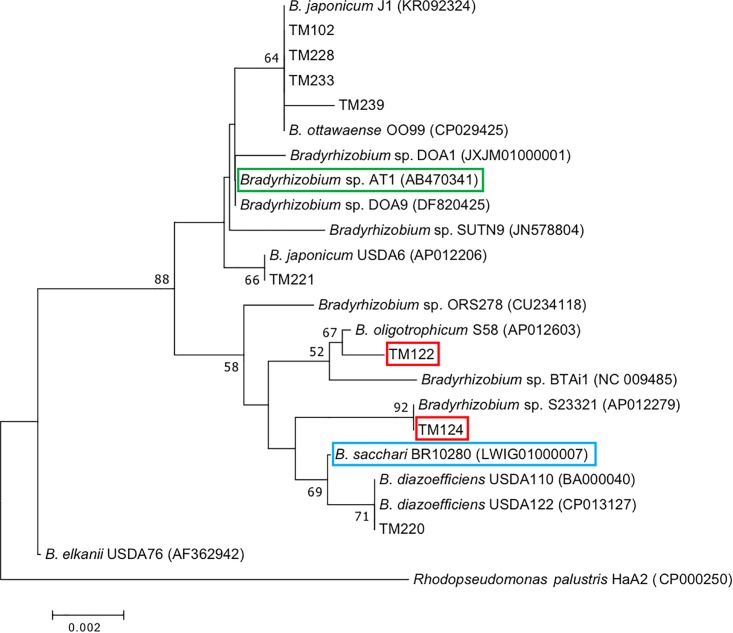
Phylogenetic tree of 16S rRNA genes of bradyrhizobial isolates from sorghum roots (prefix TM) constructed by the neighbor-joining method. Numbers at the nodes are percentages of 1000 bootstrap replications supporting the partition. Red boxes indicate functional diazotrophs suggested by our omics analysis derived from sorghum roots (TM122, TM124), while blue and green boxes indicate diazotrophic bradyrhizobia from sugarcane (BR10280) and sweet potato (AT1), respectively.

Phylogenetic analysis based on 16S rRNA gene sequences indicated that bradyrhizobial isolates TM122 and TM124 were very close to photosynthetic *B. oligotrophicum* S58^T^ (Okubo et al., 2013) and non-nodulating *Bradyrhizobium* sp. S23321 (Okubo et al., 2012a), respectively (Figure 4). The isolate TM220 apparently belonged to *B. diazoefficiens*, while the other bradyrhizobial isolates (TM102, TM228, TM233, TM239, and TM221) were close to *B. japonicum*. Both species are typical soybean endosymbionts (Kaneko et al., 2002, 2011). Metagenome and subsequent proteome analyses suggested that potential candidates of functional diazotrophs in the field-grown sorghum roots were close relatives of *Bradyrhizobium* sp. S23321 and *B. oligotrophicum* S58 (Supplementary Tables S6, S7, S9). Thus, we used the isolates TM122 and TM124 for subsequent genome analysis and N_2_ fixation experiments.

### Draft Genome Analyses of Bradyrhizobial Isolates

**FIGURE 5:**
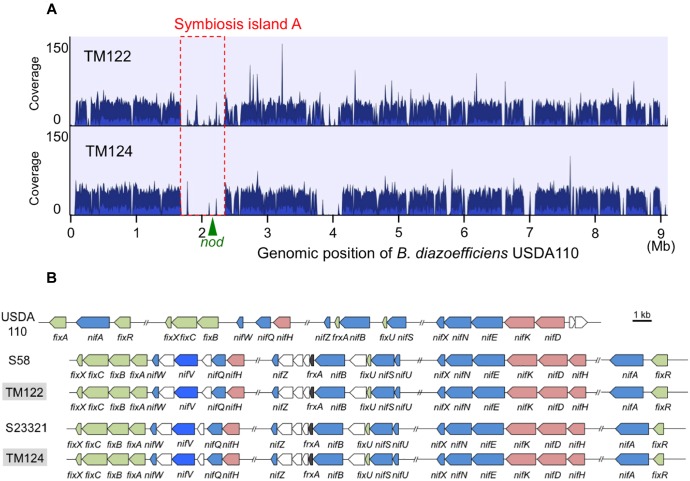
Genomic comparison between sorghum root isolates of TM122 and TM124 and phylogenetically close bradyrhizobia. **(A)** Mapping of MiSeq reads of TM122 (1,191,548 reads of 213 bp in average length) and TM124 (1,190,703 reads of 208 bp in average length) on *B. diazoefficiens* USDA110^T^ genome with symbiosis island (Kaneko et al., 2002). The coverage within symbiosis island (red dotted lines) including *nod* genes (Kaneko et al., 2002, 2011) were apparently lower than those of other genomic regions. Cluster of *nif* genes of USDA110^T^ is located on symbiosis island (Kaneko et al., 2002). **(B)** The organization of *nif* genes of TM122 and TM124 (light gray) compared to those of *B. diazoefficiens* USDA110^T^, *B. oligotrophicum* S58, and *Bradyrhizobium* sp. S23321. Colored pentagons indicate (pink) structural *nifHDK*, (blue) other *nif*, and (green) *fix* genes.

Mapping of the MiSeq reads of TM122 and TM124 to the soybean-nodulating *B. diazoefficiens* USDA110^T^ genome showed that their genomes lacked symbiosis island structures of soybean bradyrhizobia that include *nod* genes (Figure 5A). A BLAST search confirmed that the genomes of both strains lacked the common nodulation genes (*nodDYABC*) required for legume nodulation (Andrews and Andrews, 2017). After assembling the MiSeq reads of TM122 and TM124, we retrieved the sequence contigs containing *nif* genes. The organization of *nif* genes in each genome resembled those of *B. oligotrophicum* S58^T^ and *Bradyrhizobium* sp. S23321, but differed from those of nodulating *B. diazoefficiens* USDA110^T^, which contains symbiosis islands (Kaneko et al., 2002; Kaneko et al., 2011; Okubo et al., 2016).

### Acetylene Reduction Assay of Two Bradyrhizobial Isolates

The potential for N_2_ fixation by the two bradyrhizobial isolates was evaluated in free-living and plant-associated states (Table 5). Free-living cells of TM122 and TM124 in semi-solid medium produced ethylene over time in the presence of acetylene (Supplementary Figure S5) and showed significant ARA in the semi-solid medium (Table 5). This result indicates that both isolates can fix N_2_ in a free-living state.

**Table 5:** Acetylene-reducing activity (ARA) of free-living cells of *Bradyrhizobium* sp. TM122 and TM124 in semi-solid medium and sorghum seedlings inoculated with TM122 and TM124.

Inoculation	Semi-solid medium (pmol tube^-1^ h^-1^)	Sorghum seedling (pmol plant^-1^ h^-1^)	
		
		Limited N	Continuous N
TM122	64.7 ± 7.8^∗∗^	22.6 ± 4.7^∗^	31.4 ± 7.5^∗^
TM124	7810 ± 520^∗∗∗^	7.71 ± 1.48	13.8 ± 0.8
Uninoculated	1.4 ± 0.1	8.26 ± 0.96	10.1 ± 1.7


*Data show average ± standard error of three replicates. Asterisk indicates significant difference compared with uninoculated treatment (Welch’s *t*-test, ^∗^*P* < 0.05; ^∗∗^*P* < 0.01, and ^∗∗∗^*P* < 0.01). After ammonium nitrate (0.05 mM) was supplied to all sorghum pots by 7 days after inoculation, the N supply was ceased (Limited N) or continued (Continuous N).*

Thereafter, seeds from the sorghum line KM2 were inoculated with isolates TM122 or TM124 and aseptically grown for 28 days after inoculation (Supplementary Figure S3). We detected weak but significant ARA (22.6–31.4 pmol plant^-1^ h^-1^) in the roots inoculated with TM122 compared to that in uninoculated roots. In contrast, TM124-inoculated roots showed no ARA under the two different treatments of N supply under our experimental conditions (Table 5 and Supplementary Figure S3), which may have been because of weak colonization or low nitrogenase expression in the sorghum seedlings following TM124 inoculation.

## Discussion

Our omics results revealed that functional N_2_-fixing bradyrhizobia associated with the roots of field-grown sorghum plants have significant N_2_-fixing activities in late growth stages. Experimentally, in addition to the *nifH* gene encoding nitrogenase reductase (NifH), the usage of *nifDK* genes encoding NifDK facilitated the reliable identification of functional diazotrophs in sorghum roots (Figure 2), as the *nifH* gene is often diversely multiplicated (Figure 5B; Okubo et al., 2016). Here, we discuss the results in terms of the level of nitrogen fixation, phylogenetic comparison with diazotrophs in other crops, and *Bradyrhizobium* diversity in N_2_ fixation and nodulation.

The N_2_-fixing activities of the field-grown sorghum roots (102 DAT) were determined by both the ARA and ^15^N_2_-feeding methods (Tables 1, 2). The N_2_-fixing activities of the roots were consistent between the methods (Table 3), indicating that the values obtained for the N_2_-fixing activities are reliable. Wani et al. (1984) evaluated the ARA of intact sorghum plants grown in sand, soil, and farmyard manure. Although the ARA depended on the manure content, temperature, and sorghum cultivar, the average ARA values of 15 cultivars was 625 nmol C_2_H_4_ plant^-1^ h^-1^ at 30–35°C during the day. This is similar to the value of 585.8 nmol C_2_H_4_ plant^-1^ h^-1^ in the KM1 roots (Table 1), although the experiments were performed in very different environments (Supplementary Table S11).

Active expression of the dinitrogenase reductase-encoded gene (*nifH*) was abundant in sugarcane stems, sweet potato stems, and pineapple leaves, which are rich in sugars and organic acids (Yoneyama et al., 2017). The leaves and stems of field-grown sorghum showed no ARA at 28 and 71 DAT except for a low ARA value for the sorghum line KM2 (Table 1 and Supplementary Table S1). In contrast, considerably higher ARA values were detected in sorghum lines KM1, KM2, and KM4 at 71 DAT (Table 1). These results suggest that the functional N_2_-fixing bacteria exclusively reside in the roots of field-grown sorghum.

*Bradyrhizobium* are versatile bacteria inhabiting soils and plants (Zhalnina et al., 2013; VanInsberghe et al., 2015; Jones et al., 2016; Szoboszlay et al., 2017). Genomic and ecological studies have focused on soybean bradyrhizobia (*B. diazoefficiens* and *B. japonicum*) because of their prominent roles in nodule formation and their agricultural significance (Masuda et al., 2016; Shiro et al., 2016; Saeki et al., 2017; Siqueira et al., 2017). However, photosynthetic diazotrophs often nodulate an aquatic legume, *Aeschynomene*, but sometimes lack *nod* genes (Giraud et al., 2007; Okubo et al., 2012a; Okazaki et al., 2016), and, thus, appear to function as an intermediate between free-living diazotrophs and classical nodulating diazotrophs (Okubo et al., 2013). Our results suggest that sorghum roots harbor functional N_2_-fixing bradyrhizobia that are similar to photosynthetic *B. oligotrophicum* S58^T^ and *Bradyrhizobium* sp. S23321.

Recent metatranscriptome analyses targeting *nifH* genes suggested that *Bradyrhizobium* species play a role in sugarcane-associated N_2_ fixation (Thaweenut et al., 2011; Fischer et al., 2012). Representative isolates, including BR10280^T^, from surface-sterilized sugarcane tissues (Rouws et al., 2014) were recently classified as *B. sacchari* (de Matos et al., 2017). The phylogenetic position of *B. sacchari* BR10280^T^ is close to that of *B. diazoefficiens* USDA110^T^, a typical soybean-nodulating bacterium (Figure 4). *Bradyrhizobium* sp. AT1 (Terakado-Tonooka et al., 2013; Okubo et al., 2016) from sweet potato is close to the soybean-nodulating *B. japonicum* USDA6^T^ (Figure 4). Although the phylogenetic positions of eight sorghum isolates were widely distributed within *Bradyrhizobium* (Figure 4), our omics data suggest that the candidates responsible for *in planta* N_2_ fixation are close relatives of *B. oligotrophicum* S58^T^ and *Bradyrhizobium* sp. S23321 such as TM122 and TM124. Recently, diverse non-diazotrophic bradyrhizobia were endophytically colonized in *Arabidopsis thaliana* roots (Schneijderberg et al., 2018). Therefore, functional diversity may be important for symbiosis and N_2_ fixation.

Two bradyrhizobial isolates from the sorghum roots based on our omics data showed potential for N_2_ fixation (ARA), at least in sorghum seedlings inoculated with TM122 (28 days after sowing) and in the free-living state for TM122 and TM124 (Table 5). Under field conditions, no ARA was detected in the sorghum roots at 28 DAT (53 days after sowing), but the highest ARA values were observed at 102 DAT (Table 1). There are two possibilities for explaining this growth-stage dependent phenomenon. First, N fertilization depletion may explain the highest nitrogenase activity at 102 DAT under field conditions. Second, physiological factors in plant-microbe interactions were likely involved. N_2_ fixation also occurs at a high rate in the late growth stages of field-grown maize, although diazotrophic bacteria were not identified (van Deynze et al., 2018). Thus, the expression of bradyrhizobial nitrogenase may require specific environments that provide appropriate carbon source supply, chemical signals, and low oxygen concentration (Terakado-Tonooka et al., 2008; Rouws et al., 2014; Yoneyama et al., 2017) along with plant stage and microbial community development in the roots. Recently, root mucilage of land maize was found to be a suitable microenvironment for N_2_ fixation and subsequent plant assimilation of fixed nitrogen (van Deynze et al., 2018).

*Bradyrhizobium* cells often endophytically colonize plant roots (Chaintreuil et al., 2000; Okubo et al., 2012a, 2013; Piromyou et al., 2015b). *Bradyrhizobium* sp. SUT-PR9 deeply invaded the central tissues of rice roots (Piromyou et al., 2015a,b). Cells of *B. oligotrophicum* S58^T^ and *Bradyrhizobium* sp. ORS278 inhabited the surface tissues (epidermal and surface cortex cells) of cultivated and wild rice plants (Chaintreuil et al., 2000; Okubo et al., 2012b, 2013). Thus, bradyrhizobia may endophytically colonize sorghum root tissues. Microscopy studies should be conducted to localize bradyrhizobia in sorghum roots.

Interactions between diazotrophs and host plant genotypes have remained unclear in the use of N_2_-fixing bacteria in crops (Okubo et al., 2013; Yoneyama et al., 2017). Further analyses targeting bradyrhizobia and sorghum genotypes may contribute to the understanding of the interactions between N_2_-fixing bradyrhizobia and sorghum plants for sustainable agriculture.

## Conclusion

The highest nitrogen-fixing activities were detected in the roots of sorghum lines KM1 and KM2 in the late growth stage (102 DAT). Nitrogenase structural genes (*nifH, nifD*, and *nifK*) in the metagenome sequences were assigned to *Bradyrhizobium* species by metagenome analysis. Proteome analysis indicated that three NifHDK proteins of *Bradyrhizobium* species were consistently detected across sample replicates. Most of these proteins were assigned to photosynthetic *B. oligotrophicum* S58^T^ and non-nodulating *Bradyrhizobium* sp. S23321. The polyphasic approach suggested that major functional N_2_-fixing bacteria in the sorghum roots are unique bradyrhizobia that resemble non-nodulating *Bradyrhizobium* sp. S23321 and photosynthetic *B. oligotrophicum* S58^T^.

## Author Contributions

SH, TK, KY, TF, TT, and KM designed the research. SH, TM, SW, and YK analyzed the data. SH and TM analyzed the DNA sequences of PCR products and metagenome. YK performed the proteome analysis. TK, SH, and SW characterized isolated diazotrophs. SH and KM wrote the article.

## Conflict of Interest Statement

The authors declare that the research was conducted in the absence of any commercial or financial relationships that could be construed as a potential conflict of interest.

## Footnotes

1 http://www.bioinformatics.babraham.ac.uk/projects/fastqc/

2 http://www.migap.org/
