# Supplementary material for: Identification of Nitrogen-Fixing Bradyrhizobium Associated With Roots of Field-Grown Sorghum by Metagenome and Proteome Analyses
Source: Front Microbiol. 2019 Mar 12;10:407. doi: 10.3389/fmicb.2019.00407 (PMC6422874; doi:10.3389/fmicb.2019.00407)
Supplement: Supplementary file 1 [file Data_Sheet_1.PDF]

Supplemental Materials

Identification of nitrogen-fixing *Bradyrhizobium* associated with roots of field-grown sorghum by metagenome and proteome analyses

Shintaro Hara,[a] Takashi Morikawa,[a] Sawa Wasai,[a] Yasuhiro Kasahara,[b] Taichi Koshiba,[c] Kiyoshi Yamazaki,[d] Toru Fujiwara,[d] Tsuyoshi Tokunaga,[c] Kiwamu Minamisawa[a]

Graduate School of Life Sciences, Tohoku University, Sendai, Miyagi, Japan[a]; Institute of Low Temperature Science, Hokkaido University, Sapporo, Hokkaido, Japan [b]; EARTHNOTE Co. Ltd., Ginoza-son, Kunigami-gun, Okinawa, Japan [c]; Graduate School of Agricultural and Life Sciences, University of Tokyo, Tokyo[d]

Table S1. Statistical evaluation of acetylene-reducing activity (ARA) between bottles in the presence and absence of acetylene[a]

DAT	Tissue	Line	C_2H_4 production (nmols plant^{-1} h^{-1}) [a]		ARA (nmols C_2H_4 plant^{-1} day^{-1}) [b]
			with C_2H_2	without C_2H_2	
0	Seedling	KM1	0.1 ± 0.0	0.1 ± 0.0	0.0 ± 0.0
0	Seedling	KM2	0.2 ± 0.1	0.1 ± 0.0	0.1 ± 0.1
0	Seedling	KM4	0.6 ± 0.4	0.0 ± 0.0	0.5 ± 0.4
0	Seedling	KM5	0.1 ± 0.0	0.2 ± 0.1	- 0.1 ± 0.0
28	Shoot	KM1	3.0 ± 0.3	3.1 ± 0.3	- 0.1 ± 0.3
28	Shoot	KM2	2.1 ± 0.2	3.0 ± 0.4	- 0.9 ± 0.2
28	Shoot	KM4	3.2 ± 0.6	3.1 ± 0.6	0.1 ± 0.6
28	Shoot	KM5	2.4 ± 0.6	2.1 ± 0.5	0.3 ± 0.6
28	Root	KM1	0.3 ± 0.0	0.4 ± 0.0	- 0.1 ± 0.0
28	Root	KM2	0.8 ± 0.1	0.5 ± 0.1	0.2 ± 0.1
28	Root	KM4	0.5 ± 0.2	0.5 ± 0.1	0 ± 0.2
28	Root	KM5	0.3 ± 0.0	0.2 ± 0.0	0.1 ± 0.0
71	Leaf	KM1	0.9 ± 0.5	0.4 ± 0.2	0.4 ± 0.4
71	Leaf	KM2	1.6 ± 0.4	0.0 ± 0.0	1.6 ± 0.4
71	Leaf	KM4	0.5 ± 0.0	0.2 ± 0.0	0.2 ± 0.0
71	Leaf	KM5	0.5 ± 0.1	0.3 ± 0.1	0.2 ± 0.1
71	Stem	KM1	4.3 ± 0.3	2.9 ± 0.9	1.4 ± 0.6
71	Stem	KM2	2.9 ± 0.3	3.3 ± 0.8	- 0.4 ± 0.5
71	Stem	KM4	0.9 ± 0.3	1.6 ± 0.4	- 0.7 ± 0.4
71	Stem	KM5	1.1 ± 0.0	1.0 ± 0.4	0.1 ± 0.5
71	Root	KM1	41.3 ± 10.2	5.3 ± 0.9	36.1 ± 10.9*
71	Root	KM2	58.5 ± 15.2	5.9 ± 0.7	52.6 ± 15.6 *
71	Root	KM4	28.2 ± 9.6	3.1 ± 0.4	25.1 ± 9.2
71	Root	KM5	2.6 ± 0.8	0.9 ± 0.6	1.7 ± 0.7
102	Root	KM1	587.3 ± 100.2	1.5 ± 0.2	585.8 ± 100.0 **
102	Root	KM2	333.4 ± 22.0	0.9 ± 0.2	332.5 ± 21.8 **
102	Root	KM4	292.7 ± 73.4	0.6 ± 0.1	292.1 ± 73.3*
102	Root	KM5	6.7 ± 1.5	0.0 ± 0.0	6.7 ± 1.5*

[a] Data represent mean ± SE (n = 3). [b] Asterisks indicate significant differences in C_2H_2 production between with- and without acetylene groups (Welch's test, *P < 0.05; **P < 0.01). DAT indicates days after transplant.

Table S2. Statistical summary of metagenomic data obtained from the bacterial community of sorghum lines KM1 and KM2.

	Replication of each line			
Line	Replication 1	Replication 2	Replication 3	
Total number of sequence reads [a]				Total
KM1	5,346,496	5,424,150	4,386,990	15,157,636
KM2	4,876,240	5,023,450	4,319,188	14,218,878
Average sequence length (bp/read)				Average
KM1	206	203	196	202
KM2	207	208	208	208

[a] Total number of high-quality sequence reads

Table S3. Read number of nitrogenase genes (*nifHDK*) in three biological replicates of sorghum line KM1.

Class	Order	Family	Genus	KM1-1			KM1-2			KM1-3		
				nifH	*nifD*	*nifK*	*nifH*	*nifD*	*nifK*	*nifH*	*nifD*	*nifK*
Alphaproteobacteria	*Rhizobiales*	*Bradyrhizobiaceae*	*Bradyrhizobium*	12	16	11	16	11	13	11	15	13
Alphaproteobacteria	*Rhizobiales*	*Xanthobacteraceae*	*Azorhizobium*		1	1		1	3			
Alphaproteobacteria	*Rhizobiales*	*Bradyrhizobiaceae*	*Bosea*				1					
Alphaproteobacteria	*Rhizobiales*	*Phyllobacteriaceae*	*Mesorhizobium*				2	1				
Alphaproteobacteria	*Rhizobiales*	*Methylobacteriaceae*	*Methylobacterium*				1	1			1	
Alphaproteobacteria	*Rhizobiales*	*Beijerinckiaceae*	*Methylocella*		1							
Alphaproteobacteria	*Rhizobiales*	*Methylocystaceae*	*Methylocystis*									3
Alphaproteobacteria	*Rhizobiales*	*Rhizobiaceae*	*Rhizobium*		2	2	1					
Alphaproteobacteria	*Rhizobiales*	*Bradyrhizobiaceae*	*Rhodopseudomonas*									1
Alphaproteobacteria	*Rhodospirillales*	*Rhodospirillaceae*	*Rhodospirillum*								1	
Alphaproteobacteria	*Rhizobiales*	*Rhizobiaceae*	*Sinorhizobium*			1						
Alphaproteobacteria	*Sphingomonadales*	*Sphingomonadaceae*	*Sphingomonas*									1
Betaproteobacteria	*Burkholderiales*	*Comamonadaceae*	*Leptothrix*								1	
Betaproteobacteria	*Burkholderiales*	*Comamonadaceae*	*Pseudodesulfovibrio*									1
Betaproteobacteria	*Burkholderiales*	*Comamonadaceae*	*Variovorax*					2				
Gammaproteobacteria	*Enterobacteriales*	*Enterobacteriaceae*	*Klebsiella*								4	2
Gammaproteobacteria	*Enterobacteriales*	*Enterobacteriaceae*	*Kosakonia*	1		3						2
Gammaproteobacteria	*Enterobacteriales*	*Enterobacteriaceae*	*Pantoea*								1	2
Gammaproteobacteria	*Immundisolibacterales*	*Immundisolibacteraceae*	*Immundisolibacter*		1							
Gammaproteobacteria	*Pseudomonadales*	*Pseudomonadaceae*	*Azotobacter*									1
Gammaproteobacteria	*Enterobacteriales*	*Yersiniaceae*	*Rahnella*									1
Deltaproteobacteria	*Desulfovibrionales*	*Desulfovibrionaceae*	*Desulfovibrio*								3	
Bacilli	*Bacillales*	*Paenibacillaceae*	*Paenibacillus*		2							
Nostocales	*Nostocaceae*	*Anabaena*	*Anabaena*						1	2		
Cyanobacteria	*Nostocales*	*Rivulariaceae*	*Calothrix*					1				
Nostocophycideae	*Nostocales*	*Nostocaceae*	*Nostoc*						4			
Chlorobia	*Chlorobiales*	*Chlorobiaceae*	*Chlorobaculum*									3
Chlorobia	*Chlorobiales*	*Chlorobiaceae*	*Chlorobium*					2			3	
Verrucomicrobia	*Opitutales*	*Opitutaceae*	*Opitutaceae bacterium*					3			2	

Table S4. Read number of nitrogenase genes (*nifHDK*) in three biological replicates of sorghum line KM2.

Class	Order	Family	Genus	KM2-1			KM2-2			KM2-3		
				nifH	*nifD*	*nifK*	*nifH*	*nifD*	*nifK*	*nifH*	*nifD*	*nifK*
Alphaproteobacteria	*Rhizobiales*	*Bradyrhizobiaceae*	*Bradyrhizobium*	12	20	11	12	10	10	11	21	16
Alphaproteobacteria	*Rhizobiales*	*Xanthobacteraceae*	*Azorhizobium*	1		1						
Alphaproteobacteria	*Rhizobiales*	*Bradyrhizobiaceae*	*Bosea*	2								
Alphaproteobacteria	*Rhizobiales*	*unclassified Rhizobiales*	*Hartmannibacter*						1			
Alphaproteobacteria	*Rhizobiales*	*Phyllobacteriaceae*	*Mesorhizobium*				1					
Alphaproteobacteria	*Rhizobiales*	*Methylobacteriaceae*	*Methylobacterium*							2		
Alphaproteobacteria	*Rhizobiales*	*Rhizobiaceae*	*Rhizobium*	1								
Betaproteobacteria	*Burkholderiales*	*Comamonadaceae*	*Pseudacidovorax*	1								
Betaproteobacteria	*Burkholderiales*	*Comamonadaceae*	*Pseudodesulfovibrio*						1			
Gammaproteobacteria	*Enterobacteriales*	*Enterobacteriaceae*	*Klebsiella*								3	1
Gammaproteobacteria	*Immundisolibacterales*	*Immundisolibacteraceae*	*Immundisolibacter*						3			
Deltaproteobacteria	*Desulfovibrionales*	*Desulfovibrionaceae*	*Desulfovibrio*				2	1				
Nostocales	*Nostocaceae*	*Anabaena*	*Anabaena*							1		
Nostocophycideae	*Nostocales*	*Nostocaceae*	*Nostoc*				2			1		
Chlorobia	*Chlorobiales*	*Chlorobiaceae*	*Chlorobaculum*						2			
Chlorobia	*Chlorobiales*	*Chlorobiaceae*	*Chlorobium*					3				

Table S5. Incidence of *nifHDK* genes in metagenome reads of root microbiomes of sorghum lines KM1 and KM2[a].

Genus	Frequency per 10^6 reads					
	Sorghum line KM1			Sorghum line KM2		
	nifH	*nifD*	*nifK*	*nifH*	*nifD*	*nifK*
Bradyrhizobium	2.6 ± 0.4**	2.8 ± 0.7**	2.5 ± 0.5**	2.5 ± 0.1**	3.7 ± 1.5**	2.7 ± 0.9**
Azorhizobium		0.1 ± 0.1*	0.2 ± 0.3*	0.1 ± 0.1		0.1 ± 0.1
Bosea	0.1 ± 0.1			0.1 ± 0.2		
Hartmannibacter						0.1 ± 0.1
Mesorhizobium	0.1 ± 0.2	0.1 ± 0.1		0.1 ± 0.1		
Methylobacterium	0.1 ± 0.1	0.1 ± 0.1*		0.2 ± 0.3		
Methylocella		0.1 ± 0.1				
Methylocystis			0.2 ± 0.4			
Rhizobium	0.1 ± 0.1	0.1 ± 0.2	0.1 ± 0.2	0.1 ± 0.1		
Rhodopseudomonas			0.1 ± 0.1			
Rhodospirillum		0.1 ± 0.1				
Sinorhizobium			0.1 ± 0.1			
Sphingomonas			0.1 ± 0.1			
Leptothrix		0.1 ± 0.1				
Pseudacidovorax				0.1 ± 0.1		
Pseudodesulfovibrio			0.1 ± 0.1			0.1 ± 0.1
Variovorax		0.1 ± 0.2				

Genus	Frequency per 10^6 reads					
	Sorghum line KM1			Sorghum line KM2		
	nifH	*nifD*	*nifK*	*nifH*	*nifD*	*nifK*
Klebsiella		0.3 ± 0.5	0.2 ± 0.3		0.2 ± 0.4	0.1 ± 0.1
Kosakonia	0.1 ± 0.1		0.3 ± 0.3*			
Pantoea		0.1 ± 0.1	0.2 ± 0.3			
Immundisolibacter		0.1 ± 0.1				0.2 ± 0.3
Azotobacter			0.1 ± 0.1			
Rahnella			0.1 ± 0.1			
Desulfovibrio		0.2 ± 0.4		0.1 ± 0.2	0.1 ± 0.1	
Paenibacillus		0.1 ± 0.2				
Anabaena	0.2 ± 0.3		0.1 ± 0.1	0.1 ± 0.1		
Calothrix		0.1 ± 0.1				
Nostoc			0.2 ± 0.4	0.2 ± 0.2*		
Chlorobaculum			0.2 ± 0.4			0.1 ± 0.2
Chlorobium		0.4 ± 0.3*			0.2 ± 0.3	
Opitutaceae bacterium		0.3 ± 0.3*				

[a] Data are mean ± SD of three biological replicates (Tables S3, S4). Asterisks indicate that each *nif* gene was detected in **three and *two replicates.

Table S6. BLAST hit of bradyrhizobial *nifHDK* genes in metagenome reads of root microbiome of sorghum line KM1 [a]

Close relative	Accession No.	KM 1-1			KM 1-2			KM 1-3			Mean abundance (%) [b]		
		nifH	*nifD*	*nifK*	*nifH*	*nifD*	*nifK*	*nifH*	*nifD*	*nifK*	*nifH*	*nifD*	*nifK*
Bradyrhizobium sp. S23321	AP012279.1	4 (33%)	6 (38%)	3 (27%)	5 (31%)	3 (27%)	4 (31%)	6 (55%)	4 (27%)	7 (54%)	40 ± 13	31 ± 6	37 ± 14
B. oligotrophicum S58	AP012603.1	4 (33%)		3 (27%)	3 (19%)	2 (18%)	4 (31%)		2 (13%)	1 (8%)	17 ± 17	11 ± 9	22 ± 12
Bradyrhizobium sp. ORS278	CU234118.1			1 (9%)		2 (18%)			2 (13%)	1 (8%)		11 ± 9	6 ± 5
B. diazoefficiens USDA 122	CP013127.1			4 (36%)		2 (18%)	4 (31%)		1 (7%)	2 (15%)		8 ± 9	28 ± 11
Bradyrhizobium sp. BTAi1	CP000494.1		2 (13%)		5 (31%)		1 (8%)	1 (9%)			13 ± 16	4 ± 7	3 ± 4
other *Bradyrhizobium* species		4 (33%)	8 (50%)		3 (19%)	2 (18%)		4 (36%)	6 (40%)	2 (15%)	30 ± 9	36 ± 16	5 ± 9
Sum of *Bradyrhizobium* species reads		12	16	11	16	11	13	11	15	13			

[a] Number in parentheses indicates the abundance in sum of bradyrhizobial *nifHDK* genes

[b] Data represent mean ± SD (n = 3)

Table S7. BLAST hit of bradyrhizobial *nifHDK* genes in metagenome reads of root microbiome of sorghum lines KM2[a].

Close relative	Accession No.	KM 2-1			KM 2-2			KM 2-3			Mean abundance (%)[b]		
		nifH	*nifD*	*nifK*	*nifH*	*nifD*	*nifK*	*nifH*	*nifD*	*nifK*	*nifH*	*nifD*	*nifK*
Bradyrhizobium sp. S23321	AP012279.1	7 (58%)	10 (50%)	5 (45%)	6 (50%)	2 (20%)	1 (10%)	3 (27%)	5 (24%)	8 (50%)	45 ± 16	31 ± 16	35 ± 22
B. oligotrophicum S58	AP012603.1		9 (45%)	1 (9%)	2 (17%)	1 (10%)	6 (60%)	5 (45%)	3 (14%)	3 (19%)	21 ± 23	23 ± 19	29 ± 27
Bradyrhizobium sp. ORS278	CU234118.1	1 (8%)		3 (27%)	1 (8%)	1 (10%)	1 (10%)	1 (9%)	4 (19%)	2 (13%)	9 ± 0	10 ± 10	17 ± 9
B. diazoefficiens USDA 122	CP013127.1			1 (9%)		2 (20%)			1 (5%)			8 ± 10	3 ± 5
Bradyrhizobium sp. BTAi1	CP000494.1		1 (5%)				1 (10%)					2 ± 3	3 ± 6
other *Bradyrhizobium* species		4 (33%)		1 (9%)	3 (25%)	4 (40%)	1 (10%)	2 (18%)	8 (38%)	3 (19%)	26 ± 8	26 ± 23	13 ± 5
Sum of *Bradyrhizobium* species reads		12	20	11	12	10	10	11	21	16			

[a] Number in parentheses indicates the abundance in sum of bradyrhizobial *nifHDK* genes

[b] Data represent mean ± SD (n = 3)

Table S8. Total number of NifHDK peptides in root microbiomes of three replications of sorghum lines KM1 and KM2[a].

Class or Phylum	Genus	KM1-1			KM1-2			KM1-3			KM2-1			KM2-2			KM2-3		
		H	D	K	H	D	K	H	D	K	H	D	K	H	D	K	H	D	K
Alphaproteobacteria	*Bradyrhizobium*	16	5	5	5	4	3	11	8	5	7	2	1	10	12	9	6	7	17
Alphaproteobacteria	*Hartmannibacter*			1						1									2
Alphaproteobacteria	*Methylobacterium*																		1
Alphaproteobacteria	*Rhizobium*	2												1					
Alphaproteobacteria	*Rhodospirillum*		1			1									1			1	
Betaproteobacteria	*Pseudodesulfovibrio*						1												
Gammaproteobacteria	*Klebsiella*		1	3		1				1						1			3
Gammaproteobacteria	*Kosakonia*										1			5			9		1
Deltaproteobacteria	*Desulfovibrio*		1															1	
Nostocales	*Anabaena*							1			1								
Nostocales	*Calothrix*					1													
Nostocales	*Nostoc*							2											1
Chlorobia	*Chlorobaculum*											1				1			1
Verrucomicrobia	*Opitutaceae*														3				
	others	1	2		1		1		2									3	
	Total	19	10	9	6	7	5	14	10	7	9	3	1	16	16	11	15	12	26

[a] H, D, and K indicate NifH, NifD, and NifK, respectively.

Table S9. Number of bradyrhizobial NifHDK peptides in root microbiome of sorghum line KM1 and KM2[a].

Close relative	Accession No.	KM 1			KM 2		
		NifH	NifD	NifK	NifH	NifD	NifK
Bradyrhizobium sp. S23321	AP012279	5 *	5 *	6 **	11 **	4 *	7 **
B. oligotrophicum S58	AP012603	12 **	2	3 **	4 *	0	11 *
Bradyrhizobium sp. BTAi1	CP000494	0	0	0	2	1	2 *
Bradyrhizobium sp. 39S1MB	CU234118	5 **	0	0	3 *	0	0
Bradyrhizobium sp. ORS278	CP017637	0	3 *	0	0	3 **	0
B. japonicum J5	CP017637	0	2	1	0	4 *	0
Bradyrhizobium sp. Soph313	EU646533	0	2	0	0	3	0
Bradyrhizobium sp. ztm3	JQ706635	0	1	1	0	1	0
Bradyrhizobium sp. M12	KF113074	2	0	0	1	0	0
B. diazoefficiens USDA122	CP013127	0	0	1	1	0	0
B. diazoefficiens NK6	AP014685	0	0	0	0	0	1
Bradyrhizobium sp. TSA15y	AB542345	1	0	0	0	0	0
B. denitrificans LMG 8443	HM047125	0	0	0	1	0	0
B. japonicum PV1.15	KY020288	1	0	0	0	0	0
Bradyrhizobium sp. psr4	JQ706672	0	0	0	0	1	0
Multi-assigned in *Bradyrhizobium*		6	2	1	4	4	6
Sum of *Bradyrhizobium* species peptides		32	17	13	27	21	27

[a] Single and double asterisks indicate that each *nif* peptide was detected in two and three replicates, respectively.

Table S10. Isolates from bacterial cells extracted from the sorghum roots

Strain name	Accession no.	Source [a]	Isolation medium [b]
Ancylobacter sp. TM202	LC433575	KM1	HM
Ancylobacter sp. TM223	LC433584	KM1	HM
Ancylobacter sp. TM226	LC433587	KM1	HM
Arthrobacter sp. TM243	LC433596	KM2	HM
Bosea sp. TM213	LC433579	KM1	HM
Bosea sp. TM241	LC433594	KM2	HM
Bradyrhizobium sp. TM102	LC367222	KM1	NA
Bradyrhizobium sp. TM122	LC367221	KM2	NA
Bradyrhizobium sp. TM124	LC367220	KM2	NA
Bradyrhizobium sp. TM220	LC433582	KM1	HM
Bradyrhizobium sp. TM221	LC433583	KM1	HM
Bradyrhizobium sp. TM228	LC433588	KM2	HM
Bradyrhizobium sp. TM233	LC433589	KM2	HM
Bradyrhizobium sp. TM239	LC433593	KM2	HM
Deinococcus sp. TM129	LC433572	KM2	NA
Deinococcus sp. TM133	LC433573	KM2	NA
Deinococcus sp. TM138	LC433574	KM2	NA
Mesorhizobium sp. TM238	LC433592	KM2	HM
Mycobacterium sp. TM205	LC433576	KM1	HM
Mycobacterium sp. TM209	LC433577	KM1	HM
Mycobacterium sp. TM211	LC433578	KM1	HM
Mycobacterium sp. TM215	LC433580	KM1	HM
Mycobacterium sp. TM218	LC433581	KM1	HM
Mycobacterium sp. TM234	LC433590	KM2	HM
Mycobacterium sp. TM242	LC433595	KM2	HM
Sphingobium sp. TM236	LC433591	KM2	HM
Terrabacter sp. TM224	LC433585	KM1	HM
Terrabacter sp. TM225	LC433586	KM1	HM

[a] Isolation source; bacterial cell extracted from root of KM1 and KM2 at 102 days after transplant.
[b] NA and HM media indicate 1/100-strength nutrient agar plate and 1/100-strength HM agar plate, respectively. The media were supplemented with 10 mL L^{-1} polymixin B.

Table S11. Temperature during incubation in N_2 fixation assay (°C)[a].

DAT	Average	Minimum	Maximum
27	23.1	21.6	25.9
72	25.8	23.7	29.2
102	26.4	23.3	28.8

[a] Temperature around incubation vials were logged every min.

At 0 days after transplant (DAT), seedlings were incubated at 25°C.

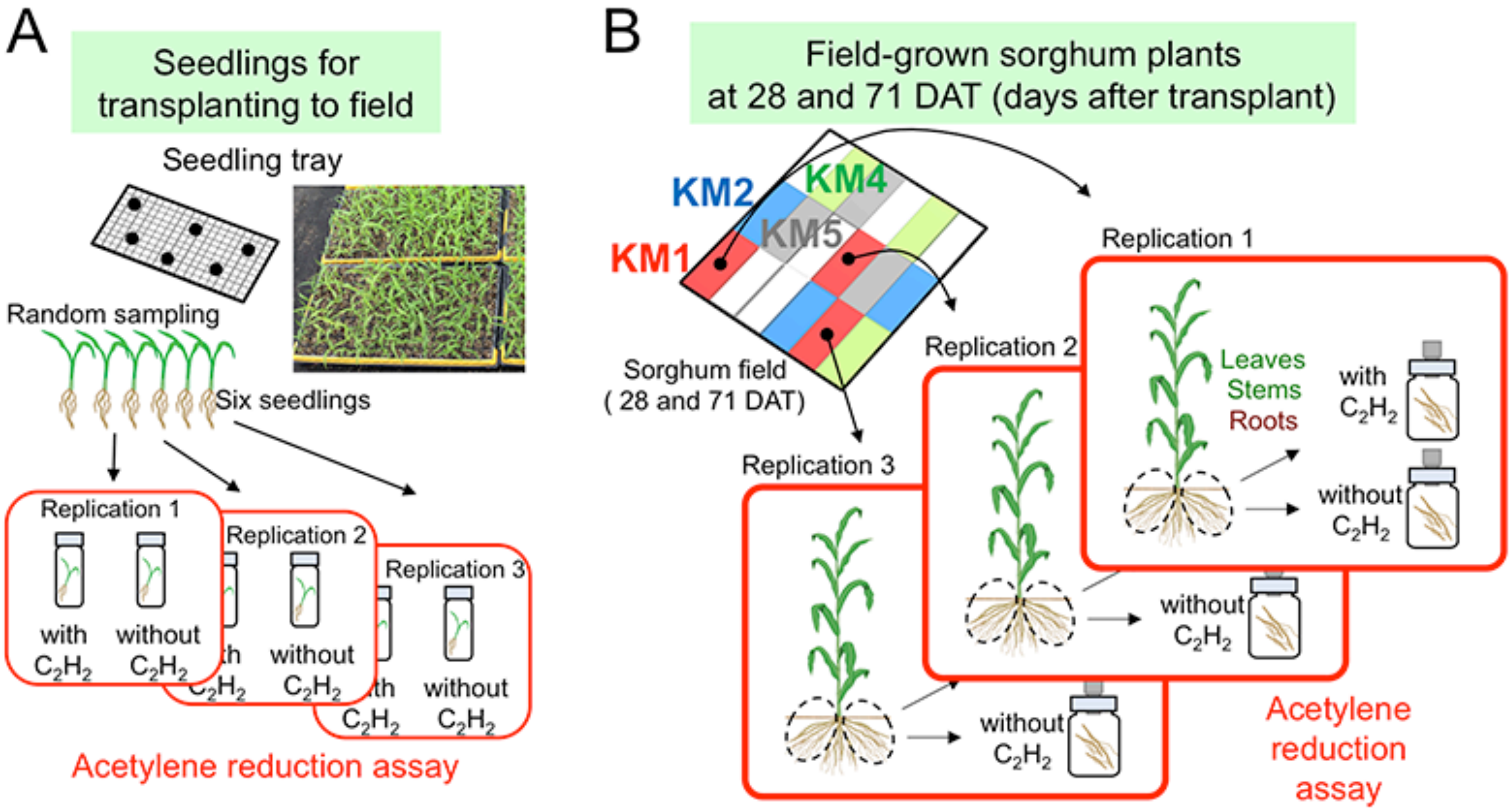

Figure S1. Outline of experimental design of acetylene reduction assay of sorghum plants at growth stages of 0, 28, and 71 days after transplant (DAT). (A) For sorghum seedlings at 0 DAT, six seedlings were randomly selected from the plug trays, which were subjected to acetylene reduction assay. (B) At 28 and 71 DAT, field-grown sorghum plants were harvested from three plots individually, and each plant tissue including the leaves, stems, and roots were subjected to acetylene reduction assay. Because ethylene is a plant hormone, ethylene production in similar plant tissues was determined with and without acetylene, where a significant increase in acetylene-dependent ethylene production was regarded as positive acetylene-reducing activity (Table S1).

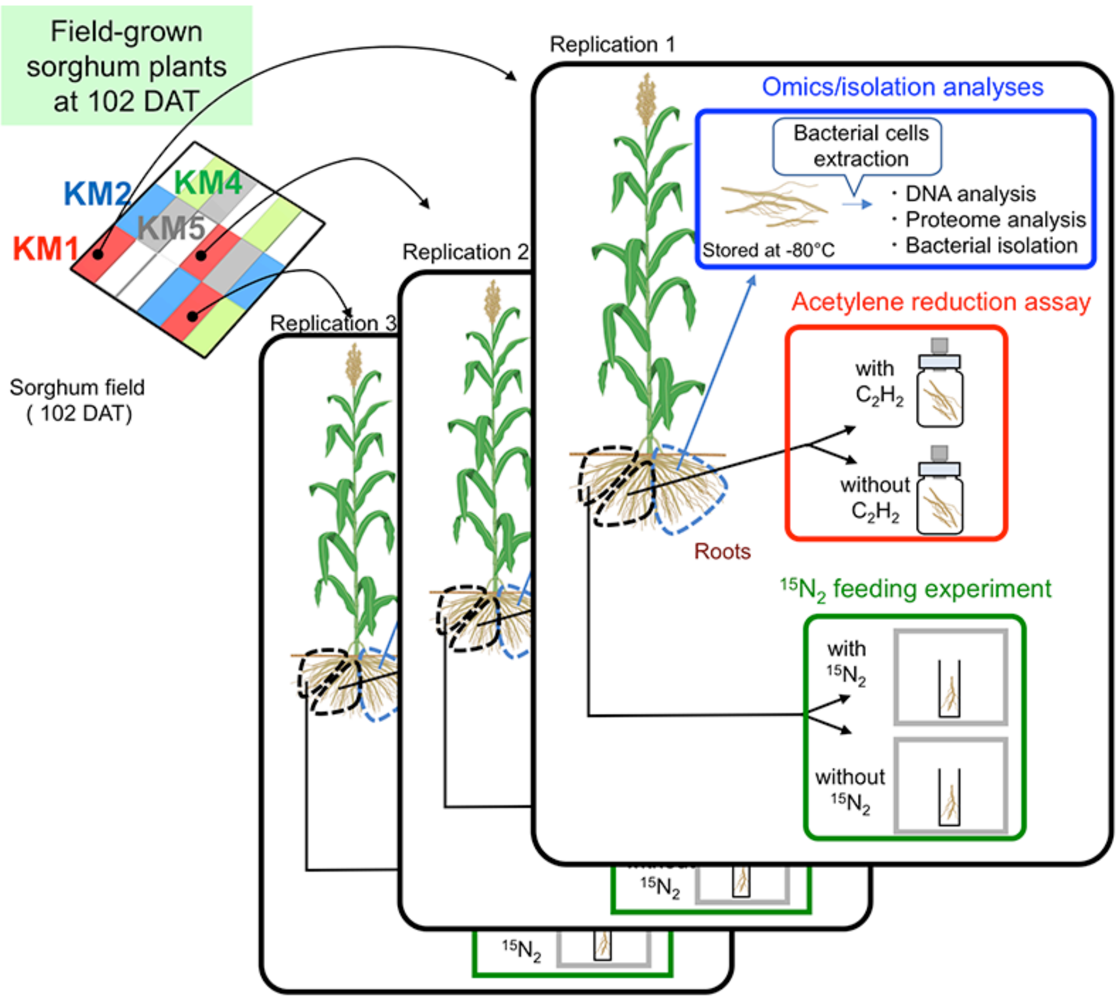

Figure S2. Outline of experimental design of acetylene reduction assay, $^{15}N_2$-feeding experiment and omics/isolation analyses at 102 days after transplant (DAT). Ethylene production of similar plant tissues was determined with and without acetylene, where a significant increase in acetylene-dependent ethylene production was regarded as positive acetylene-reducing activity (Table S1).

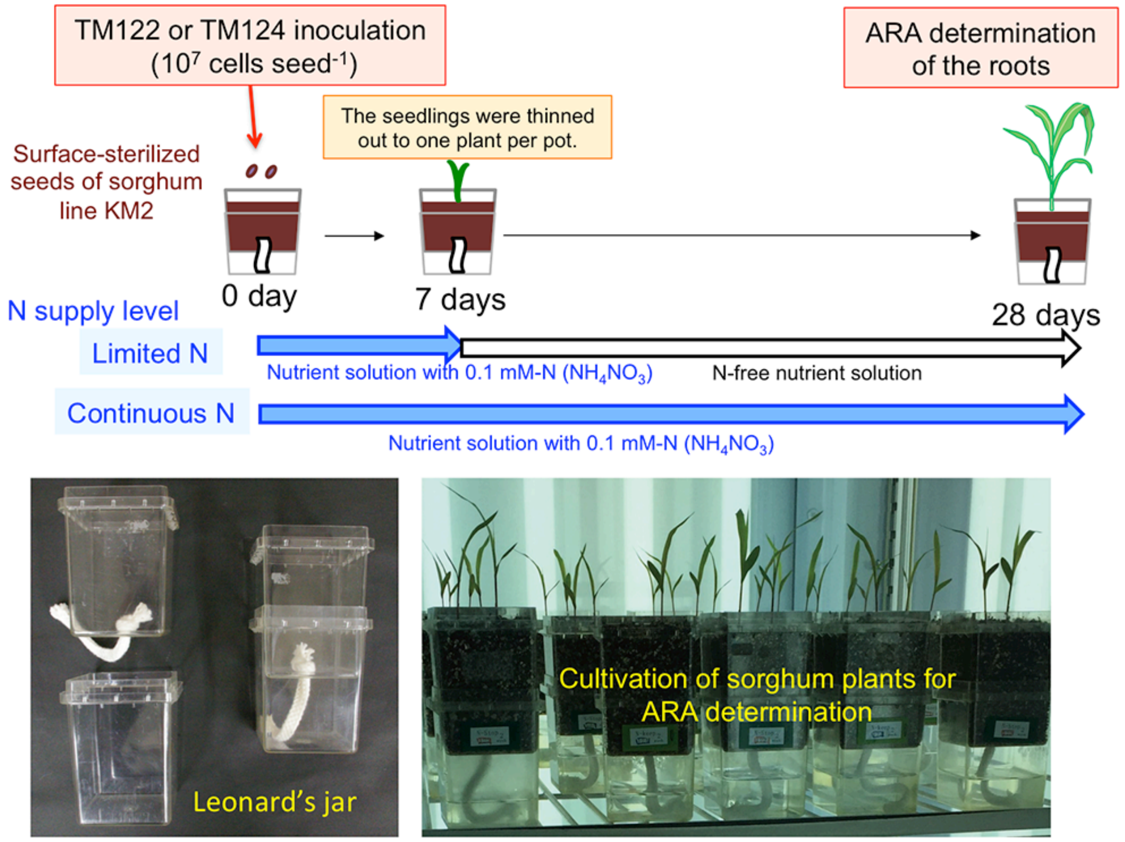

Fig. S3. Inoculation experiment of bradyrhizobial isolates to sorghum seeds

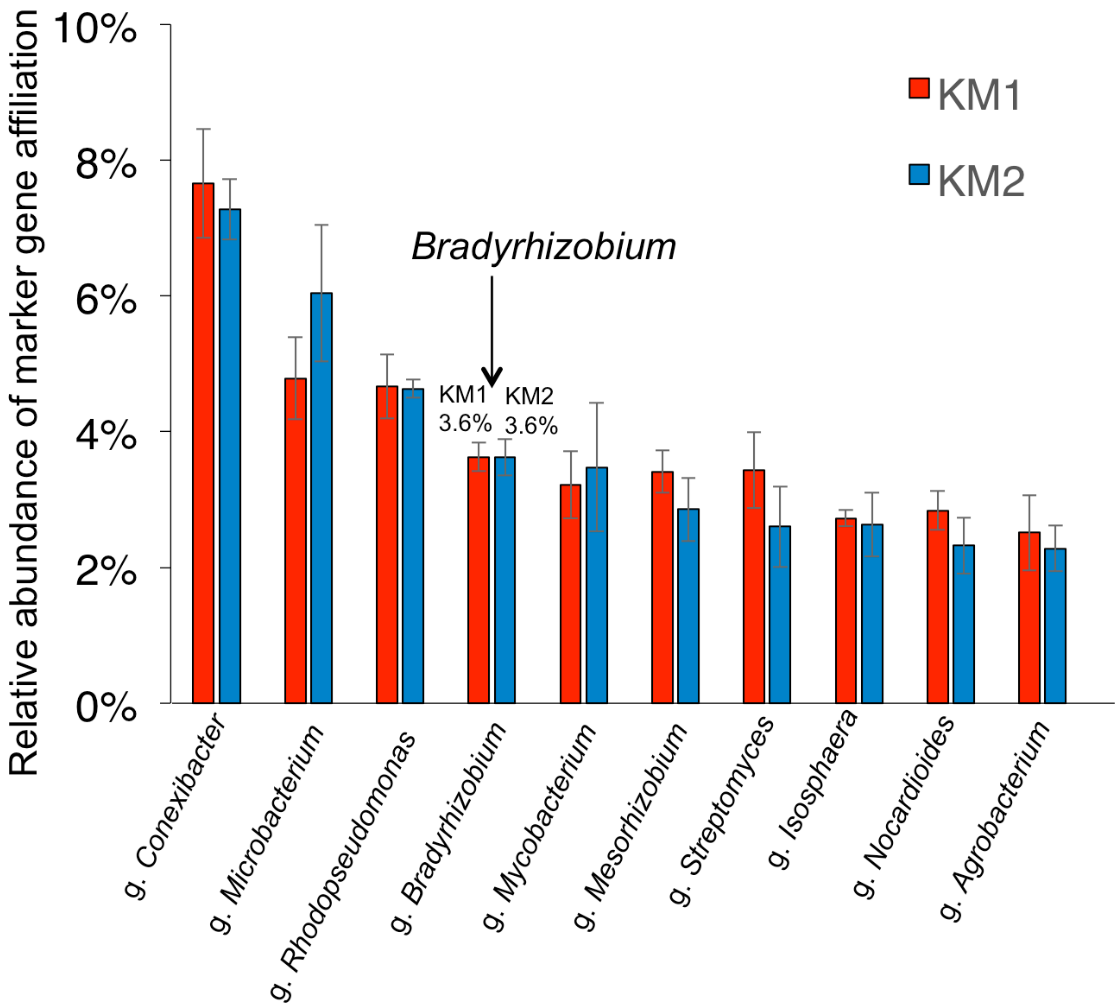

Fig. S4. Phylogenetic analysis of metagenomic data sets based on 31 bacterial marker genes. Y-axis indicates ratio of the marker gene affiliation to bacterial phyla in the metagenomic data of KM1 and KM2. Ten taxonomic groups showing higher relative abundance values are shown with standard error (bar). The numbers of marker genes extracted from KM1 and KM2 metagenome sequences were 4469 ± 333 and 3937 ± 184, respectively.

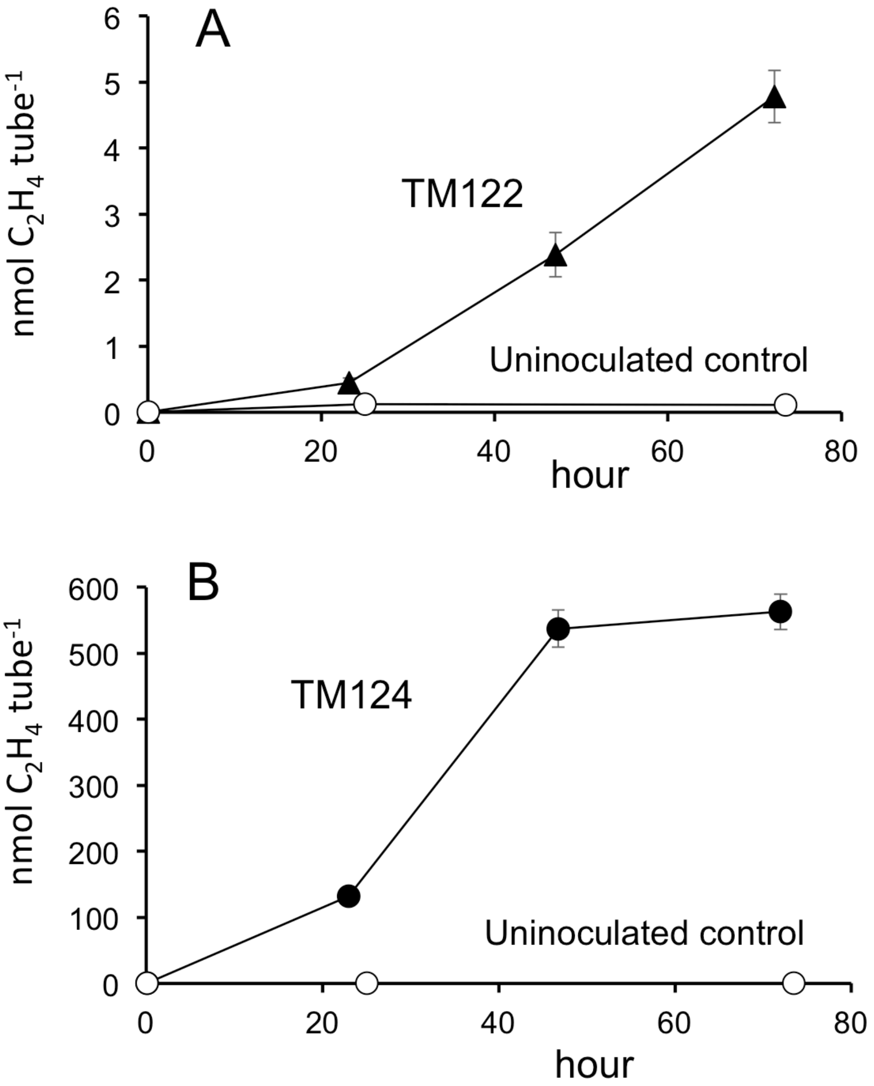

Fig. S5. Acetylene-reducing activity (ARA) of free-living cells of *Bradyrhizobium* sp. TM122 (A) and TM124 (B) in semi-solid medium. Time-course of ethylene (C_2H_2) concentrations was monitored in the headspace of test tubes in the presence of 10% (vol/vol) acetylene. Inoculation with TM122 and TM124 increased C_2H_2 concentrations over time, whereas no such increase was observed in uninoculated semi-solid medium (Uninoculated control). Data represent the average of three biological replications with standard error (bar).
